# Preparation of a Ni–ascorbic acid MOF as a recyclable catalyst for the synthesis of sulfoxides and tetrazoles†

**DOI:** 10.1039/d5ra00355e

**Published:** 2025-05-12

**Authors:** Mostafa Koolivand, Zahra Moradi, Mohsen Nikoorazm, Arash Ghorbani-Choghamarani

**Affiliations:** a Department of Chemistry, Faculty of Science, Ilam University Ilam Iran m.nikorazm@ilam.ac.ir; b Department of Organic Chemistry, Bu-Ali Sina University 6517838683 Hamedan Iran a.ghorbani@basu.ac.ir arashghch58@yahoo.com

## Abstract

This study introduces a novel, cost-effective, and environmentally friendly approach for synthesizing a heterogeneous Ni–ascorbic acid metal–organic framework (Ni–ascorbic acid MOF) catalyst *via* a hydrothermal method. The catalyst was prepared by combining nickel nitrate (Ni(NO_3_)_2_·6H_2_O) and ascorbic acid (C_6_H_8_O_6_) in DMF. Comprehensive characterization of the synthesized Ni–ascorbic acid MOF was performed using FT-IR, XRD, EDX, BET, SEM, TGA/DSC, and AAS techniques. These analyses revealed that the catalyst exhibits a spherical microsphere morphology with high crystallinity, a specific surface area of 16.62 m^2^ g^−1^, a pore diameter of 19.52 nm, and excellent thermal stability. The catalytic performance of Ni–ascorbic acid MOF was investigated in two distinct reactions, including the selective oxidation of sulfides to sulfoxides and the synthesis of 5-substituted 1*H*-tetrazoles. Under optimized reaction conditions, the catalyst demonstrated high efficiency with product yields ranging from moderate to excellent across various substrates. Furthermore, the catalyst exhibited remarkable recyclability, maintaining its activity over five consecutive cycles without significant leaching of nickel species, as confirmed by hot filtration tests. These findings underscore the potential of a Ni–ascorbic acid MOF as a sustainable and robust catalyst for diverse organic transformations.

## Abstract 2

This study introduces a novel, cost-effective, and environmentally friendly approach for synthesizing a heterogeneous Ni–ascorbic acid metal–organic framework (Ni–ascorbic acid MOF) catalyst *via* a hydrothermal method.

## Graphical abstract

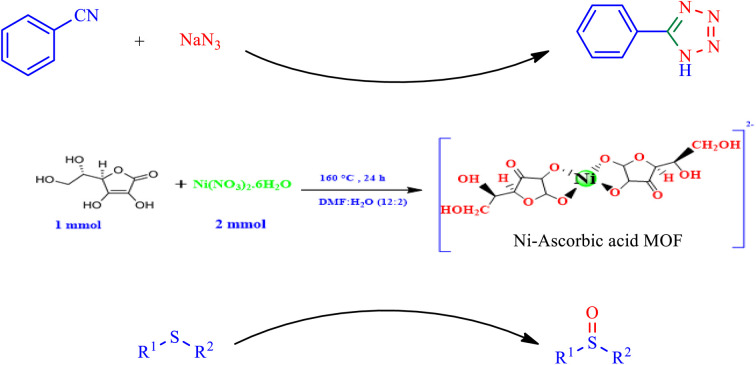

## 1. Introduction

Catalysts can be broadly categorized into two groups based on the reaction phase they occupy. Homogeneous catalysis systems exist in the same phase as the reactants, whereas heterogeneous catalysis systems operate in a different phase. The advantages of homogeneous systems include high selectivity and high yields. Conversely, heterogeneous catalysts offer several benefits, including ease of separation, which makes them environmentally friendly. They also exhibit low preparation costs, high activity, extraordinary selectivity, outstanding stability, and excellent recyclability.^1–6^ Metal–organic frameworks (MOFs) have attracted significant interest in science and technology as heterogeneous catalysts due to their exceptional catalytic activity and high surface area-to-volume ratio.^5–8^ Recently, metal–organic framework (MOF) synthesis has garnered significant attention. These frameworks are constructed by interconnecting inorganic nodes with organic ligands, resulting in highly porous materials. MOFs have been extensively studied due to their wide range of applications, such as storage, separation, and catalysis. Furthermore, their potential in biomedical applications and as sensor materials is being actively explored.^9,10^ Green chemistry, often referred to as sustainable chemistry, is a scientific discipline focused on designing chemical products and processes that minimize or eliminate the use and generation of hazardous substances. It emerged as a response to the Pollution Prevention Act of 1990 and is guided by twelve principles that emphasize waste prevention, atom economy, and the use of safer chemicals and renewable feedstocks. Unlike traditional approaches that address pollution after its creation, green chemistry seeks to prevent pollution at its molecular level by integrating sustainability into every stage of a chemical's life cycle from design to disposal. This approach not only reduces environmental harm but also enhances human health, conserves resources, and supports economic efficiency. By fostering innovative solutions, green chemistry plays a vital role in addressing global challenges such as climate change, resource depletion, and biodiversity loss while promoting safer industrial practices. For this reason, a lot of research has been done in this field. Recent advancements in metal–organic frameworks (MOFs) and nanoparticle catalysts demonstrate significant progress in sustainable chemical synthesis and detection technologies. A Hf(iv)-based MOF (CSMCRI-KNC) exhibits dual functionality as a sensitive sensor for the explosive FOX-7 and a recyclable catalyst for Knoevenagel condensation, operating under eco-friendly ethanol conditions with high atom economy.^11^ Another study also highlights the use of nickel-based metal–organic frameworks (Ni-MOF-74) combined with ionic liquids ([bmim]Br) as a catalyst system, which operates under mild conditions to achieve high selectivity and performance. This approach addresses key challenges in traditional oxidation methods, including difficulties in catalyst recycling, equipment corrosion, and environmental pollution, by using heterogeneous catalysts and environmentally benign ionic liquids.^12^ A specific approach to green chemistry has been developed through the development of a sustainable method for the oxidation of benzylic alcohols to carbonyl compounds. The use of atmospheric oxygen as an oxidant, which is abundant, inexpensive, and environmentally benign, is significant. This is in line with the principles of green chemistry that advocate the use of safer and more sustainable reagents. The use of mesoporous nickel nanoparticles supported on alumina as a heterogeneous catalyst is highlighted.^13^

In another study, the principles of green chemistry in the biogenic synthesis of silver nanoparticles (AgNPs) using the aqueous peel extract of *Punica granatum* L. are discussed. This method avoids hazardous reagents and toxic solvents and is in line with environmentally friendly methods. Furthermore, AgNPs were used as catalysts for the internal hydroxylation of aryl boronic acids to phenols under mild conditions using hydrogen peroxide as a green oxidant. This study emphasizes the role of plant-derived biomolecules in reducing environmental impacts while increasing the efficiency of nanoparticle synthesis and catalytic applications.^14^ Furthermore, the principles of green chemistry are embodied through the development of a photocatalytic system for the synthesis of aldehydes from benzyl alcohol. The use of the CdS/Ni-MOF74 composite increases the efficiency of hydrogen production and oxidation of benzyl alcohol and shows the potential to create value-added products alongside green energy in the form of H_2_.^15^ Also, sustainable synthesis methods extend to bis(indolyl)methane using β-cyclodextrin hydrate in aqueous media, achieving metal-free catalysis with minimal waste.^16^ Finally, the development of efficient protocols for organic transformations, particularly catalytic transfer hydrogenation (CTH) using hydrogen-donating solvents, is discussed, focusing on Meerwein–Ponndorf–Verley (MPV) reduction. This study introduces a Hf(iv)-based UiO-66 MOF (CSMCRI-KNC’) as a reusable heterogeneous catalyst for hydrogen transfer reactions between carbonyl compounds and alcohols, which exhibits high yields, conversions, broad substrate scope, and stability in MPV reduction.^17^ These innovations collectively advance green chemistry through recyclable catalysts, benign solvents, and energy-efficient processes.

Nickel-based catalysts are widely utilized to accelerate various chemical reactions, and these processes have become standard techniques for evaluating the reactivity of nickel species as potential catalysts. Consequently, extensive efforts have been devoted to developing diverse heterogeneous nickel catalysts for multicomponent processes. However, challenges such as leaching from catalyst supports and agglomeration of nickel-active species are commonly encountered, leading to a decline in catalytic activity during prolonged use.^18–20^ To optimize the availability of nickel species and minimize leaching, current research focuses on developing heterogeneous catalysts by securely anchoring nickel species onto suitable support materials. These support materials not only stabilize the active species and ensure uniform dispersion but also modify the electronic structure of the active sites through interfacial interactions, thereby directly influencing catalytic activity. Additionally, reusability remains a critical attribute of heterogeneous catalysts. The exceptional properties of nickel-based mesoporous structures and their application in catalytic processes, particularly for the transformation of organic functional groups, represent a significant area of ongoing research.^21–23^

Tetrazoles are synthetic heterocyclic compounds characterized by an unsaturated 5-membered aromatic ring, comprising four nitrogen atoms and one carbon atom.^7,24–32^ During recent decades, the development of catalytic methods for the synthesis of tetrazoles has attracted significant attention due to their broad applications, as reviewed by Dömling *et al.*.^8^ Tetrazoles are widely utilized in pharmaceutical chemistry, materials science, and energetic applications, making their efficient synthesis a critical area of research.

A sulfoxide is a sulfur-containing molecule characterized by two S–C bonds and an oxygen atom double-bonded to the sulfur atom. Dimethyl sulfoxide is a well-known example of a sulfoxide, commonly used as an organic solvent.^6,33–35^ A variety of synthetic and natural medications incorporate the diaryl-sulfoxide moiety, which is utilized in the treatment of numerous diseases.^36,37^ With advancements in synthesis methods, research on sulfoxide derivatives has become increasingly efficient, diverse, and accessible, enabling broader exploration and application in various fields.^37^ Furthermore, it is widely acknowledged that the most effective method for synthesizing sulfoxides involves the oxidation of sulfides using various oxidants under catalytic conditions.^2,38–43^ Despite significant advancements, there remains considerable scope for developing highly efficient methodologies utilizing MOF materials for the synthesis of sulfoxides *via* oxidation methods. In line with our interest in the synthesis and applications of MOF-based catalysts, this article proposes the design and synthesis of an innovative heterogeneous Ni–ascorbic acid MOF. This novel catalyst is evaluated for its effectiveness in the production of 5-substituted 1*H*-tetrazoles and its moderate yet efficient catalytic performance in the oxidation of sulfides to their corresponding sulfoxide derivatives.

## 2. Experimental

### 2.1. Synthesis of Ni–ascorbic acid MOF

**Scheme 1:**
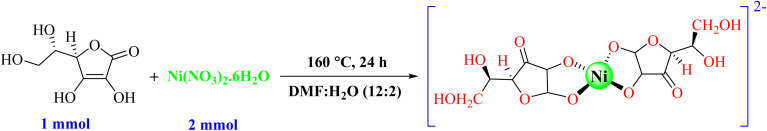
The procedure for the synthesis of Ni–ascorbic acid MOF.

The Ni–ascorbic acid MOF was synthesized using the hydrothermal method. Initially, C_6_H_8_O_6_ (1 mmol) was dissolved in H_2_O (5 mL), and then Ni(NO_3_)_2_·6H_2_O (2 mmol) dissolved in DMF (20 mL) was added. The mixture was stirred for 20 minutes, followed by heating at 160 °C for 24 hours in an autoclave. Subsequently, the Ni–ascorbic acid MOF was isolated, washed with EtOH, and sonicated for 20 minutes. Finally, it was dried at 60 °C (Scheme 1).

In the hydrothermal synthesis of a Ni–ascorbic acid MOF, DMF serves multiple critical roles. DMF acts as a polar aprotic solvent, effectively solubilizing the metal precursor and organic linker while facilitating their interaction under hydrothermal conditions. DMF influences the dielectric properties of the reaction medium, which impacts the solubility of growing MOF crystals and extends the metastable phase window for crystalline product formation. Additionally, DMF may act as a structure-directing agent by occupying specific coordination sites on metal nodes, thereby guiding the spatial arrangement of organic linkers during framework assembly. The solvent's ability to dynamically exchange with other ligands (*e.g.*, carboxylate groups) ensures the structural flexibility necessary for defect repair and crystallite growth.

### 2.2. Synthesis of sulfoxides catalyzed by Ni–ascorbic acid MOF

The reaction was initiated by mixing a catalytic amount of Ni–ascorbic acid MOF (7 mg) with sulfide (1 mmol) and hydrogen peroxide (0.4 mL), followed by stirring at room temperature without additional solvent. The reaction progress was monitored using thin-layer chromatography (TLC). Once complete, the Ni–ascorbic acid MOF was removed, and the resulting sulfoxides were extracted with ethyl acetate. The ethyl acetate extract was then dried over Na_2_SO_4_ (1.5 g), and the pure products were isolated after evaporation of the solvent.

### 2.3. Synthesizing of tetrazoles catalyzed by Ni–ascorbic acid MOF

A mixture consisting of NaN_3_ (1.3 mmol) and aryl nitrile (1 mmol) was dissolved in DMF (2 mL) in the presence of a catalytic amount of Ni–ascorbic acid MOF (30 mg). The reaction mixture was then stirred at 120 °C, with progress monitored by thin-layer chromatography (TLC). Upon completion, hydrochloric acid (4 N, 10 mL) was added, and the Ni–ascorbic acid MOF was separated from the reaction mixture. The resulting tetrazole products were isolated by extraction with ethyl acetate, dried over Na_2_SO_4_ (1.5 g), and purified by evaporation of the ethyl acetate solvent.

## 3. Results and discussion

### 3.1. Structural analysis

**Fig. 1:**
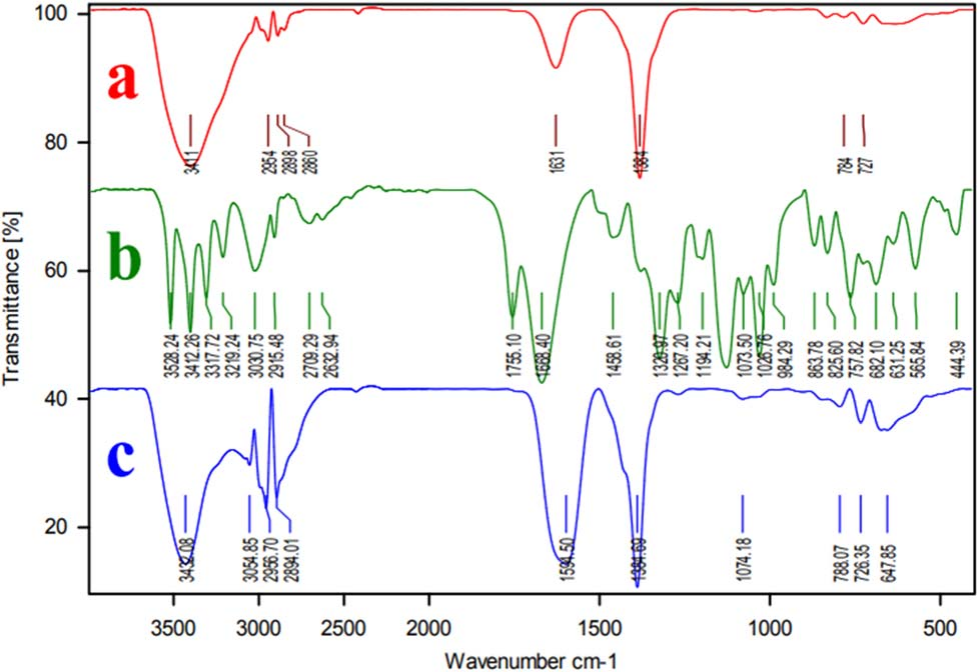
FT-IR spectra of (a) Ni(NO_3_)_2_, (b) ascorbic acid, and (c) Ni–ascorbic acid MOF.

Fourier transform infrared spectroscopy (FT-IR) was employed to analyze the materials (Fig. 1). Ascorbic acid's FT-IR spectra showed absorption bands at 1751 and 1703 cm^−1^, corresponding to the C=O bonds vibration of COOH groups (Fig. 1b). The produced Ni–ascorbic acid MOF's spectra did not show these absorption peaks, while the spectra of the Ni–ascorbic acid MOF presented two peaks for the vibration of the C=O bond vibration of COOH groups at 1560 cm^−1^. The shift of the carbonyl vibration from 1668 cm^−1^ in the pure ascorbic acid to 1560 cm^−1^ in Ni–ascorbic acid MOF indicated the metal coordination, confirming the complexation reaction between Ni and Ascorbic acid (Fig. 1c). Additionally, the most significant peaks in the area of 2922–2999 cm^−1^ can be characteristic of the C–H vibrations.^44^ The organic ligands used in Ni–ascorbic acid MOF persisted and weren't destroyed, as evidenced by the indication of aliphatic C–H vibration.^45^ Additional peaks were those for Ni–O bonding at 400 to 1000 cm^−1^ and carboxylic acid's OH at 3000 to 3600 cm^−1^. It was proposed that an intermediate compound of ascorbic acid, water, and metal ions makes up the as-prepared Ni–ascorbic acid MOF. The FT-IR results showed that Ni–ascorbic acid MOF made from nickel nitrate has distinct sharp peaks and confirmed the high degree of crystallinity of the Ni–ascorbic acid MOF.

**Fig. 2:**
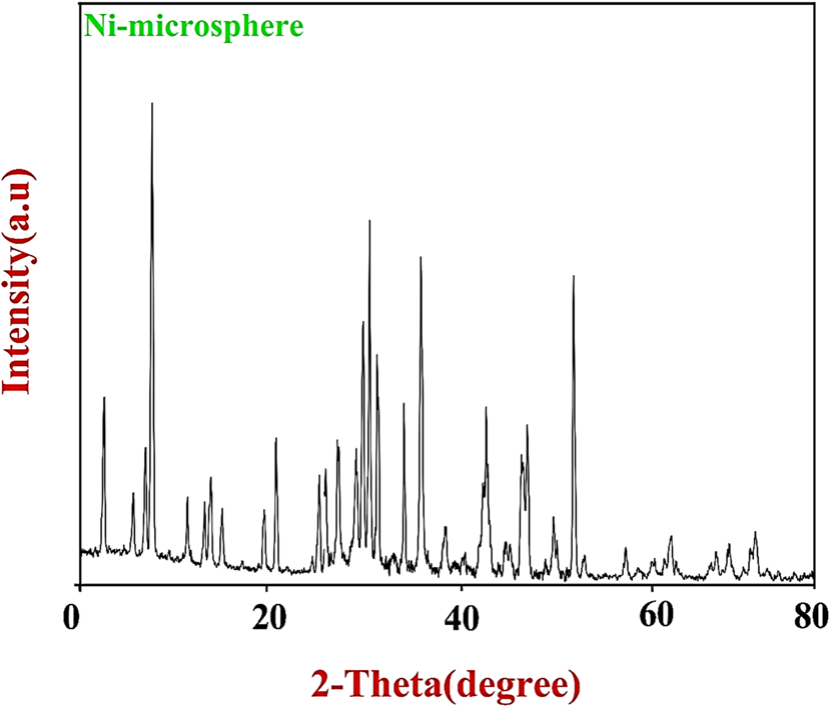
XRD pattern of Ni–ascorbic acid MOF.

The synthesized Ni–ascorbic acid MOF was characterized using X-ray diffraction (XRD) analysis. The XRD pattern of the Ni–ascorbic acid MOF, depicted in Fig. 2, displayed peaks at 2*θ* = 33.2°, 37°, 55°, and 66.2°, which were indexed to the (110) and (200) planes. These peaks may be attributed to the microsphere phase of nickel.

**Fig. 3:**
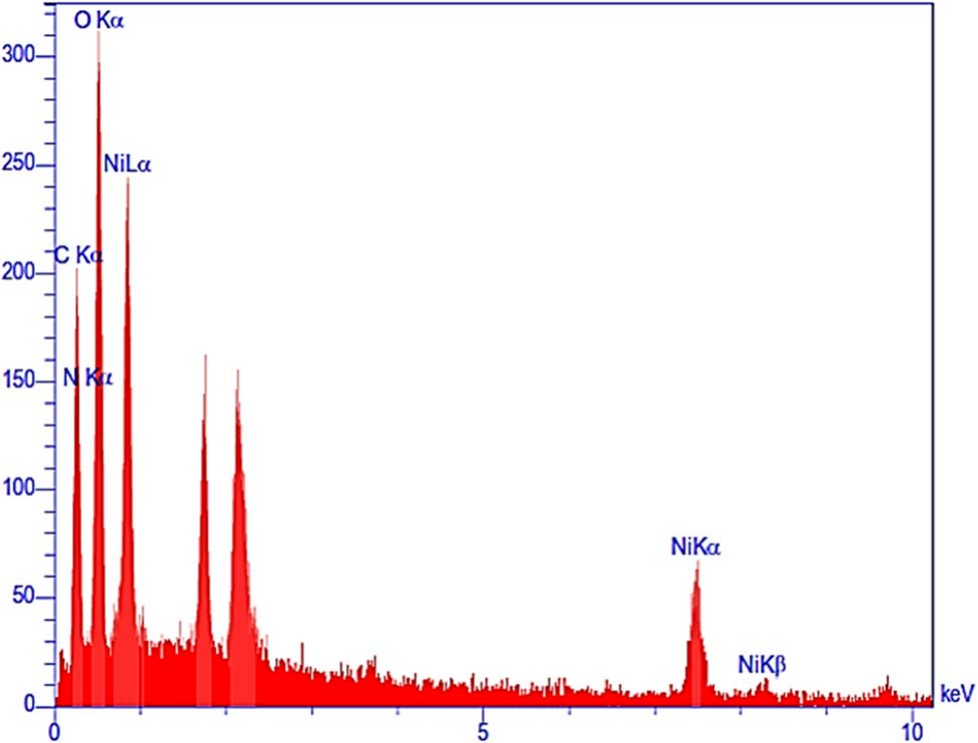
EDX analysis of Ni–ascorbic acid MOF.

The energy-dispersive X-ray (EDX) spectrum revealed the presence of nickel (3.68 wt%), oxygen (48.74 wt%), carbon (32.50 wt%), and nitrogen (13.29 wt%) in the Ni–ascorbic acid MOF catalyst, as shown in Fig. 3. These results confirm the high purity of the synthesized catalyst.

**Fig. 4:**
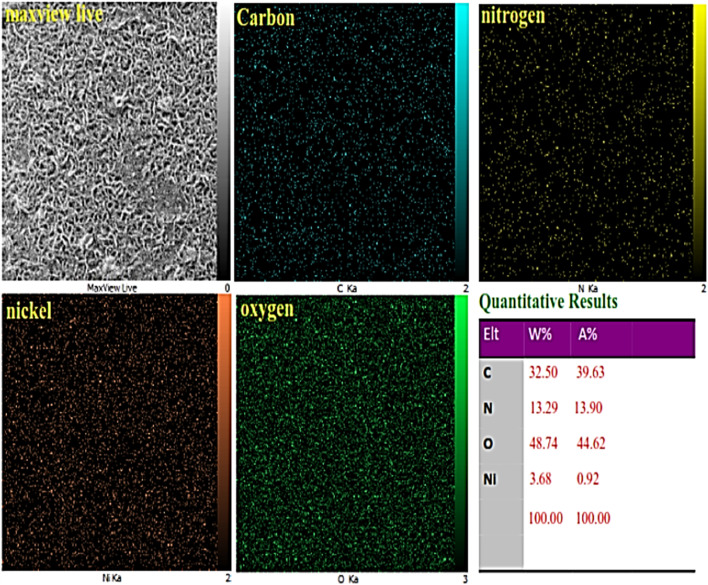
Elemental mapping analysis of Ni–ascorbic acid MOF.

The elemental mapping of the Ni–ascorbic acid MOF, as depicted in Fig. 4, confirmed the uniform distribution of carbon, oxygen, nitrogen, and nickel. The presence of the ascorbic acid scaffold, acting as a ligand, was evident from the similar distribution patterns of carbon, oxygen, and nitrogen elements. The X-ray mapping images also revealed a consistent distribution of the nickel element. Based on these findings, it can be inferred that the ascorbic acid scaffold and nickel have coordinated equally, resulting in a consistent integration of the catalytically active centers.

**Fig. 5:**
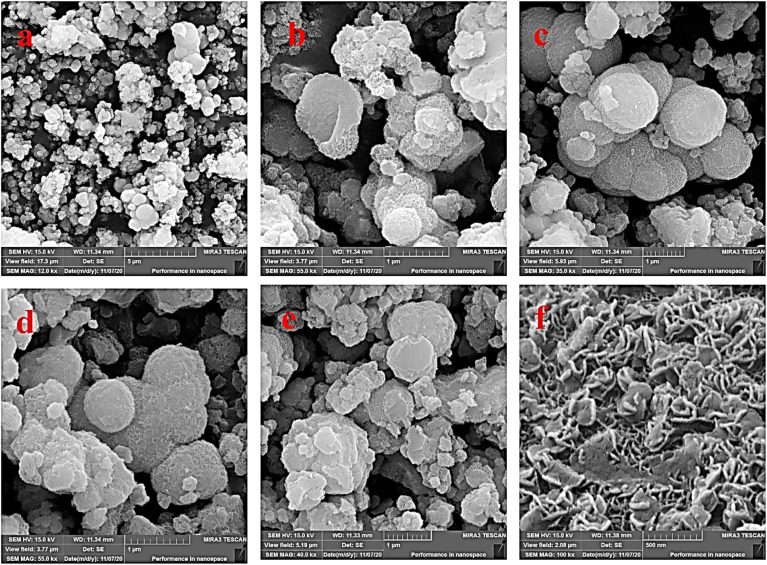
(a–f) FE-SEM images of Ni–ascorbic acid MOF.

The morphology and porosity of the Ni–ascorbic acid MOF microspheres were evaluated using field emission scanning electron microscopy (FE-SEM), with the results shown in Fig. 5. Images Fig. 5a–f present the microspheres at different magnifications. Specifically, images Fig. 5b, c, and f demonstrate that the synthesized microspheres possess a spherical shape accompanied by a snake-like morphology. These microspheres can be classified as small-scale particles, such as nanospheres or nanocapsules, based on their structural features.

**Fig. 6:**
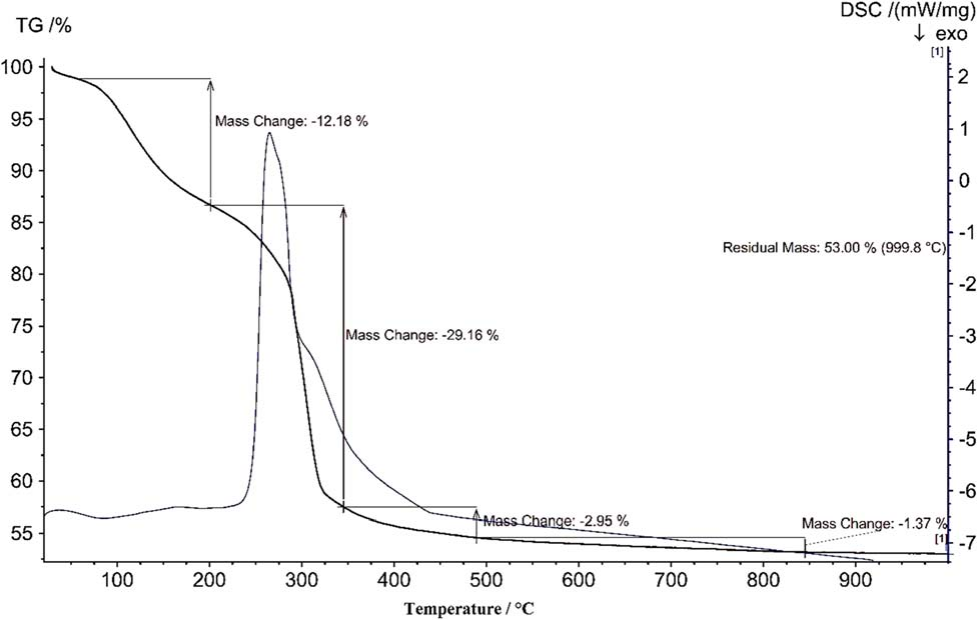
TGA and DSC craves Ni–ascorbic acid MOF.

Thermogravimetric analysis (TGA) and Differential scanning calorimetry (DSC) were employed to evaluate the thermal stability of the Ni–ascorbic acid MOF (Fig. 6). The decomposition temperature of the Ni–ascorbic acid MOF causes one severe exothermic peak process to begin at 250 °C and end at 450 °C, as seen by the DSC curve. The TGA analysis indicates two continuous weight losses (≈30% and 3%) in the region of 220–350 °C and 350–500 °C that result from organic molecules breaking down.

**Fig. 7:**
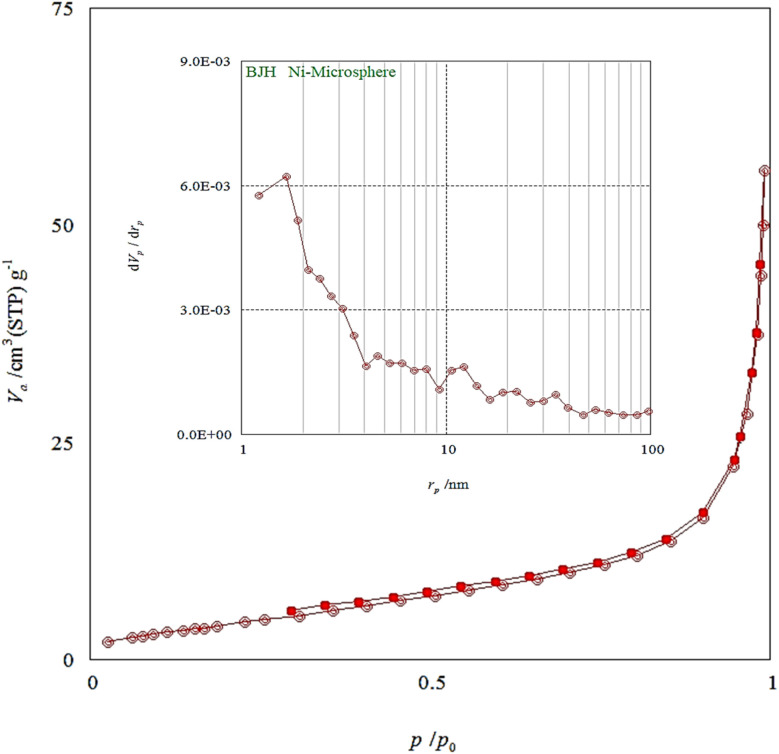
N_2_ adsorption/desorption isotherms of the Ni–ascorbic acid MOF.

Fig. 7 depicts the N_2_ adsorption–desorption isotherms of the Ni–ascorbic acid MOF and Table 1 provides a summary of the Ni-textural microsphere's characteristics. This catalyst's pore volumes, specific surface areas, and pore diameters are 0.08117 cm^3^ g^−1^, 16.628 m^2^ g^−1^, and 19.527 nm, respectively.

**Table 1:** Textural properties of Ni–ascorbic acid MOF

*S*_BET_ (m^2^ g^−1^)	Pore diameter (nm)	Pore volume (cm^3^ g^−1^)
16.628	19.527	0.08117

## 4. Catalytic study

After the successful synthesis and characterization of the Ni–ascorbic acid MOF, its catalytic activity was evaluated for sulfoxidation reactions and the preparation of tetrazoles. Methylphenyl sulfide was selected as a model substrate to optimize reaction conditions in the presence of Ni–ascorbic acid MOF. The reaction was tested in various solvents, including CH_3_CN, EtOH, CH_2_Cl_2_, and EtOAc, as well as under solvent-free conditions (Table 2, entries 1–5). The results indicated that the yield was significantly poor when the reaction was conducted in these solvents (Table 2, entries 1–4). However, a solvent-free reaction at ambient temperature using Ni–ascorbic acid MOF demonstrated successful conversion (Table 2, entry 5). Additionally, the effect of catalyst loading on product yield was investigated. It was determined that 25 mg of Ni–ascorbic acid MOF was the optimal amount for efficient conversion (Table 2, entry 6). To further explore the unique catalytic properties of Ni–ascorbic acid MOF for selective oxidation of sulfides to sulfoxides, control experiments were conducted without the catalyst. These tests yielded only trace amounts of the product (Table 2, entry 8), highlighting the critical role of Ni–ascorbic acid MOF in facilitating this transformation.

**Table 2:** Effect of different amounts of nickel ascorbic acid MOF on the oxidation of methyl phenyl sulfide

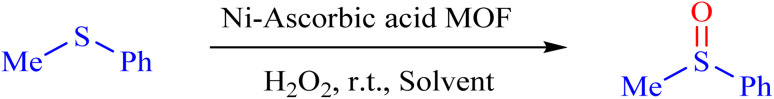				
Entry	Solvent	Catalyst (mg)	Time (min)	Yield^a^ (%)
1	EtOH	20	40	55
2	CH_3_CN	20	40	45
3	CH_2_Cl_2_	20	40	30
4	EtOAc	20	40	65
5	Solvent-free	20	40	92
6	Solvent-free	25	35	95
7	Solvent-free	10	35	75
8	Solvent-free	—	35	Trace

a Isolated yield.

In continuation, the scope of the sulfoxidation reaction spread out for the aliphatic and aromatic sulfides. As Table 3 shows, all sulfoxides were formed with reasonable yields and times.

**Table 3:** Synthesis of sulfoxides catalyzed by Ni–ascorbic acid MOF

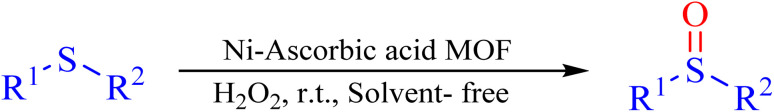						
Entry	Substrate	Product	Time (min)	Yield^a^ (%)	Melting point (°C)	
					Measured	Ref.
1	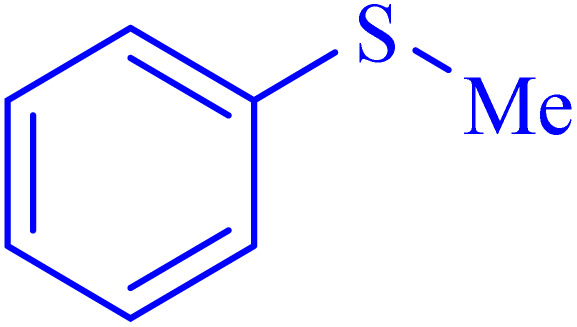	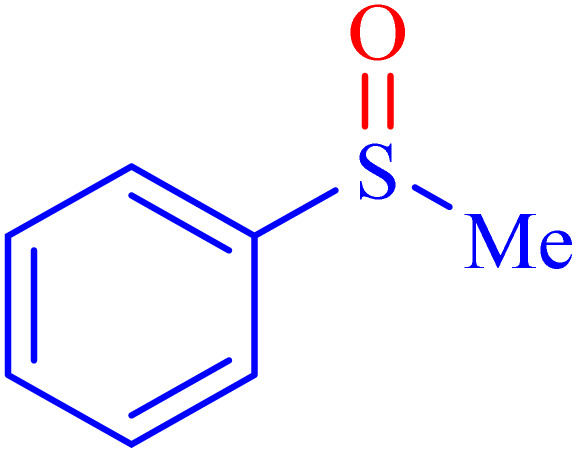	35	95	32–35	33–34 (ref. 46)
2	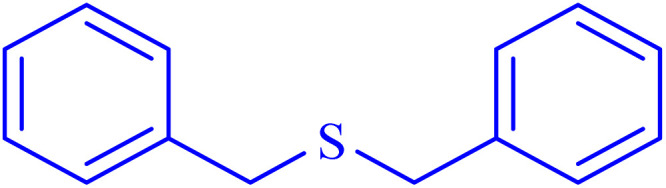	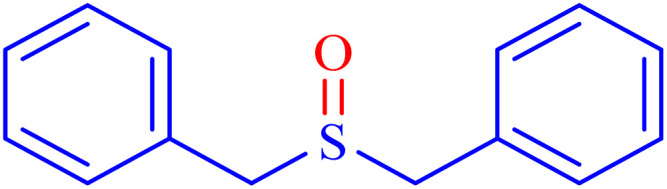	30	85	130–132	130–132 (ref. 46)
3	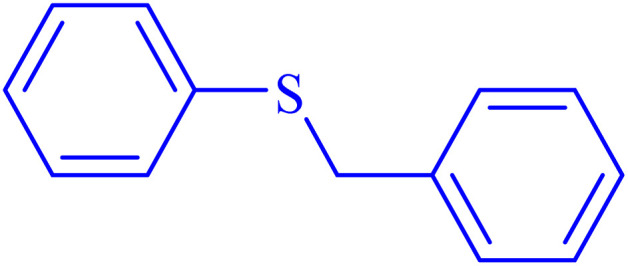	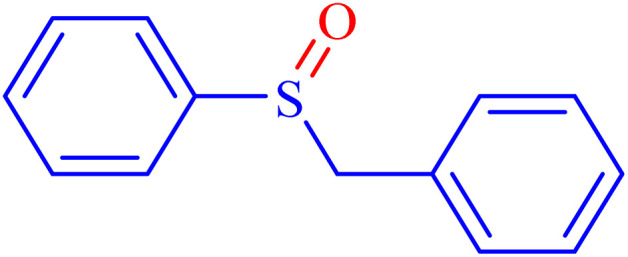	55	75	116–120	114–115 (ref. 46)
4	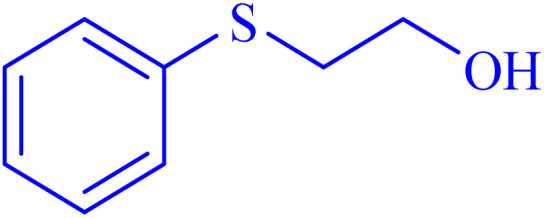	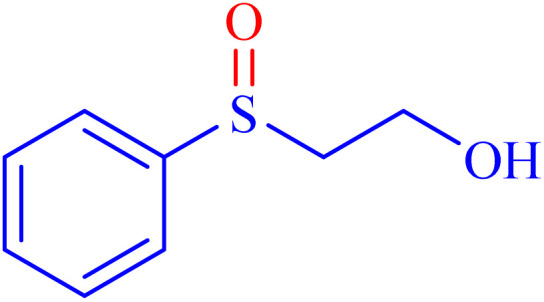	30	90	Oil	—
5	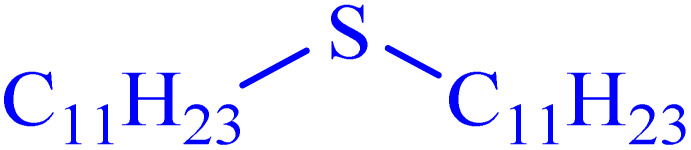	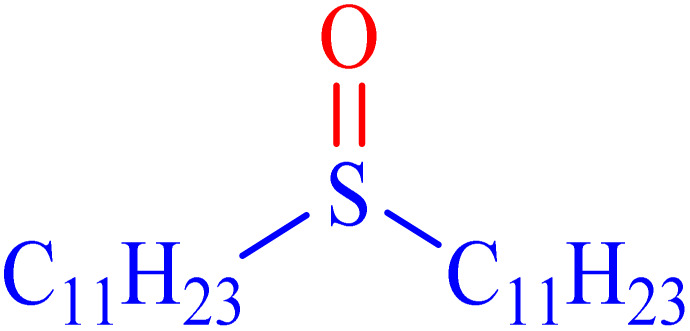	85	85	85–89	84–87 (ref. 47)
6	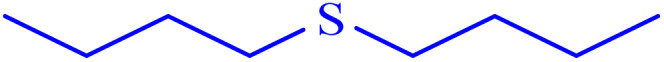	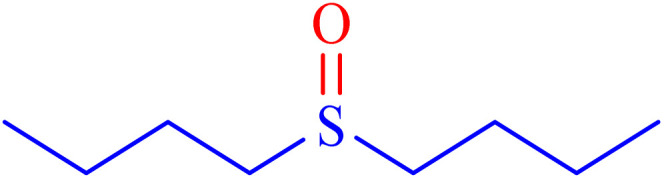	35	84	Oil	—
7	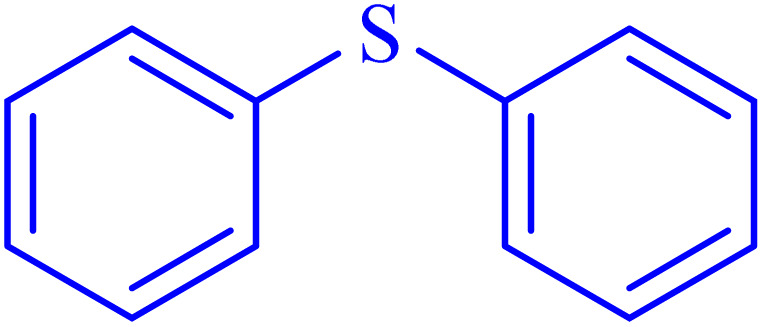	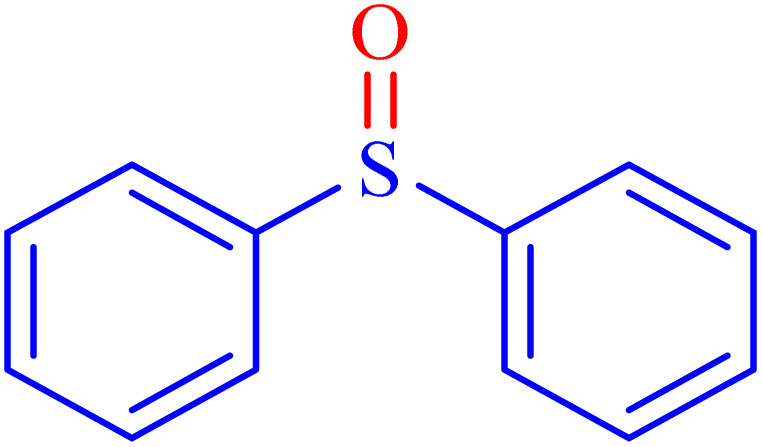	280	68	67–68	70–72 (ref. 46)

a Isolated yield.

**Scheme 2:**
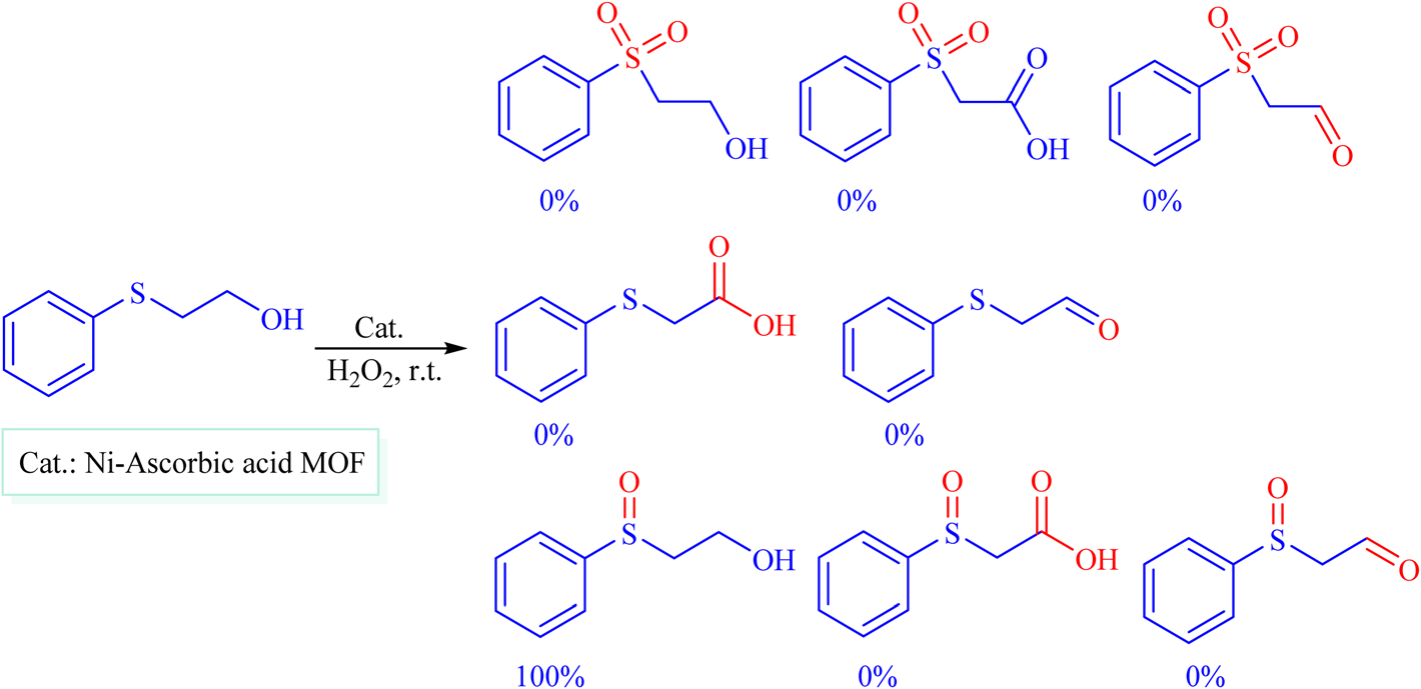
Chemoselective sulfoxidation of 2-(phenylthio)ethanol.

Selectivity is a critical factor from both industrial and laboratory perspectives. Scheme 2 demonstrates the chemoselectivity of the Ni–ascorbic acid MOF catalyst. Notably, the hydroxyl group, which could potentially compete in the oxidation reaction, remained intact during the oxidation of the sulfide moiety (Table 3, entry 4). This selectivity ensured that the reaction proceeded without the formation of unwanted byproducts, such as sulfones.

## 5. Proposed mechanism for the synthesis of sulfoxides

**Scheme 3:**
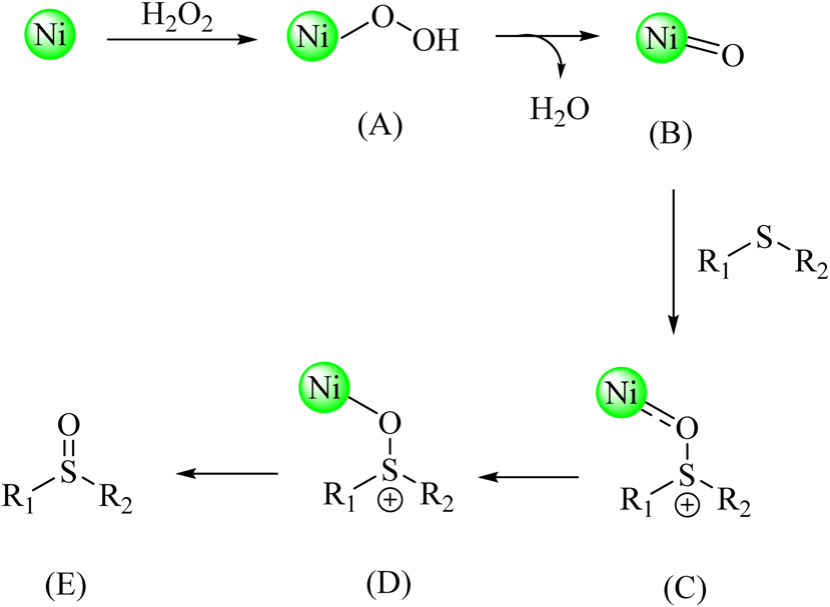
Proposed mechanism for the synthesis of sulfoxides catalyzed by Ni–ascorbic acid MOF.

The proposed mechanism for the oxidation of sulfides is shown in Scheme 3. First, hydrogen peroxide approaches the Ni–ascorbic acid MOF, forming intermediate A. After water is removed, intermediate B is formed. Next, electron transfer from the sulfide to the catalyst occurs (C). This electron transfer weakens the sulfur-carbon bonds and initiates sulfide oxidation (C, D). Finally, the oxidized sulfide product is released from the nickel center (E), and the catalyst is recovered.

In the next part, an efficient and environmentally sustainable domino protocol is reported for the synthesis of tetrazoles, including the two-component addition of aryl nitrile compounds and NaN_3_ in green media using a Ni–ascorbic acid MOF catalyst. Initially, the reaction of NaN_3_ and benzonitrile was tested as the model reaction to explore the possibility of the suggested synthetic method.

The model reaction was investigated under different conditions, including different amounts of catalyst, solvents, and temperatures. First, the catalyst-free condition was tested in DMF at 120 °C, and no product was obtained after 48 h (Table 4, entry 1). Catalyst amounts from 5–30 mg in DMF were then tested at 120 °C (Table 4, entries 2–7), and the best result was obtained with 30 mg of catalyst (Table 1, entry 7). Increasing the catalyst amount to 40 mg did not affect the yield (Table 4, entry 8). The model reaction was tested with the optimum amount of catalyst in DMSO, EtOH, H_2_O, PEG-400, and solvent-free conditions (Table 4, entries 9–13), and a decrease in efficiency was observed in each experiment. Also, to investigate the effect of temperature, the model reaction was tested at room temperature and different temperatures (Table 4, entries 14–18). At room temperature, negligible efficiency was obtained (Table 4, entry 14), and at each step, higher efficiency was obtained with increasing temperature (Table 4, entries 15–18).

**Table 4:** Optimization of the reaction conditions for the synthesis of tetrazoles catalyzed by Ni–ascorbic acid MOF

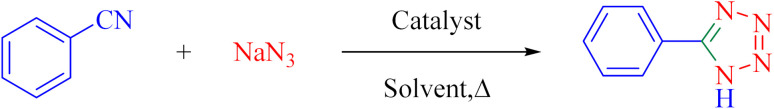				
Entry	Cat. (mg)	Solvent	Temp./°C (time/h)	Yield^a^ (%)
1	—	DMF	120 (48)	N.R
2	5	DMF	120 (8)	31
3	10	DMF	120 (8)	55
4	15	DMF	120 (8)	63
5	18	DMF	120 (8)	82
6	25	DMF	120 (8)	91
7	30	DMF	120 (8)	97
8	40	DMF	120 (8)	97
9	30	DMSO	120 (8)	62
10	30	EtOH	Reflux (8)	23
11	30	H_2_O	Reflux (8)	10
12	30	PEG-400	Reflux (2.5)	Trace
13	30	Solvent-free	120 (8)	N.R^b^
14	30	DMF	r.t (8)	Trace
15	30	DMF	40 (8)	49
16	30	DMF	80 (8)	67
17	30	DMF	100 (8)	86
18	30	DMF	110 (8)	93

a Isolated yield.

b No reaction.

The optimized synthesis strategy was further expanded to include reactions between NaN_3_ and various nitriles bearing electron-withdrawing or electron-donating groups, leading to the synthesis of heterocyclic tetrazoles, as summarized in Table 5.

**Table 5:** Synthesis of tetrazoles catalyzed by Ni–ascorbic acid MOF in DMF at 120 °C

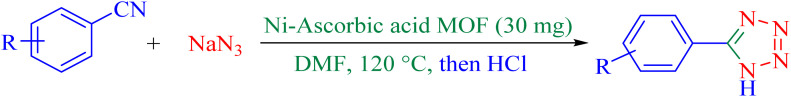						
Entry	Aryl nitrile	Product	Time (h)	Yield^a^ (%)	Melting point (°C)	
					Measured	Ref.
1	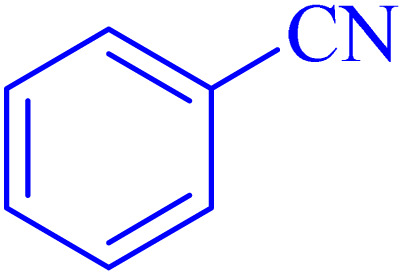	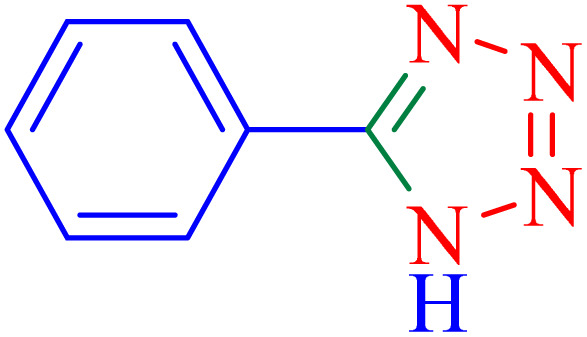	8	97	214–216	212–215 (ref. 48)
2	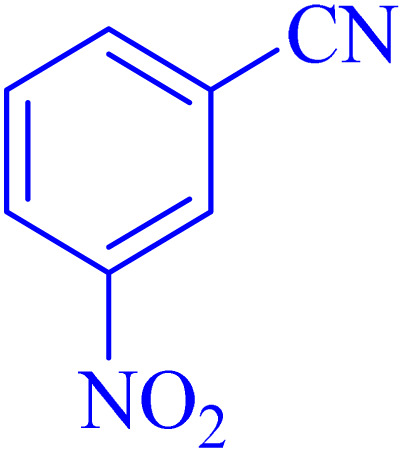	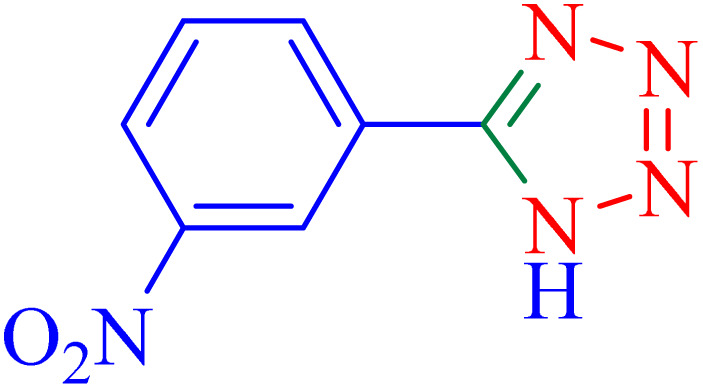	13	93	149–152	150–153 (ref. 49)
3	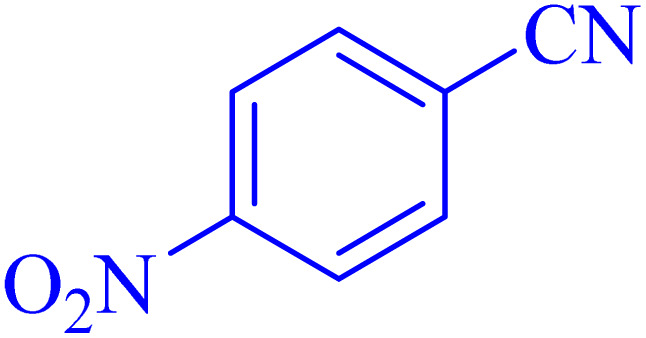	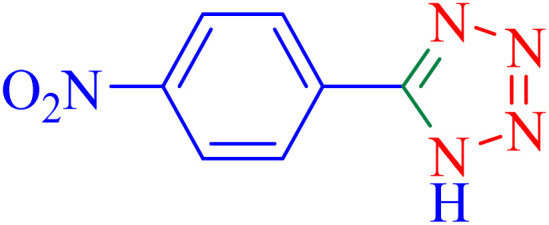	24	96	217–219	217–219 (ref. 48)
4	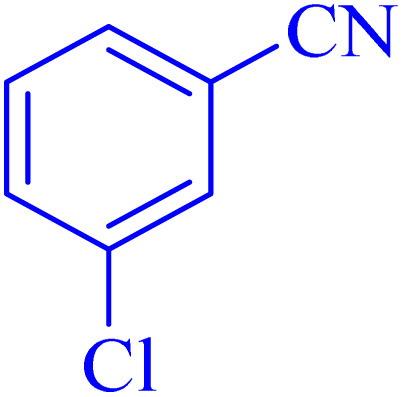	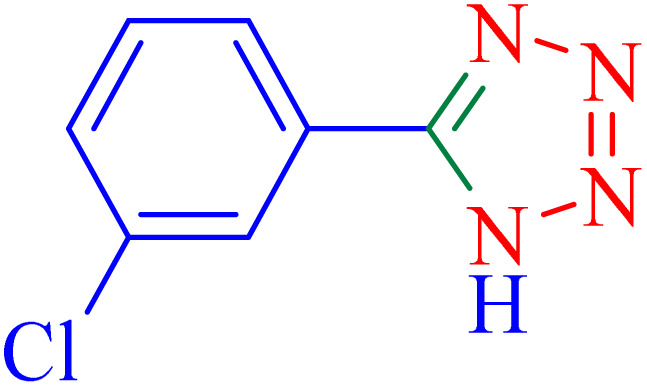	10	94	129–131	129–132 (ref. 7)
5	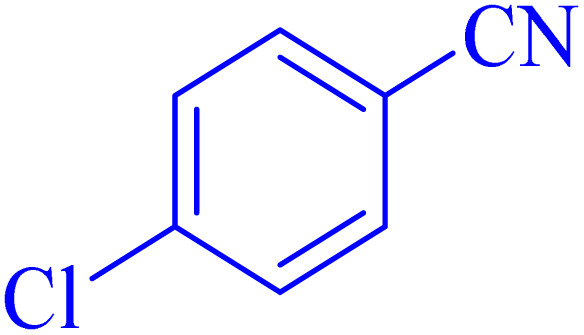	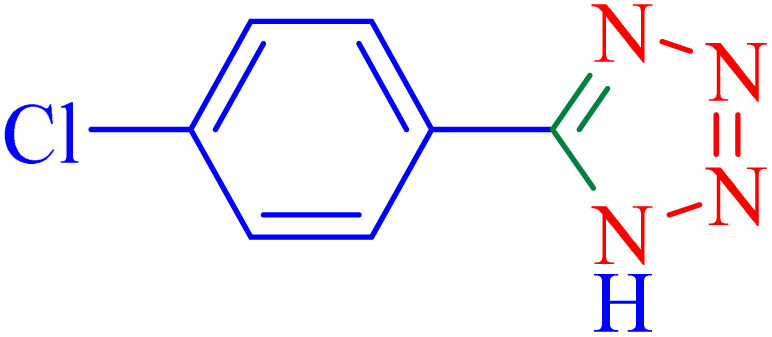	14	91	261–264	260–263 (ref. 48)
6	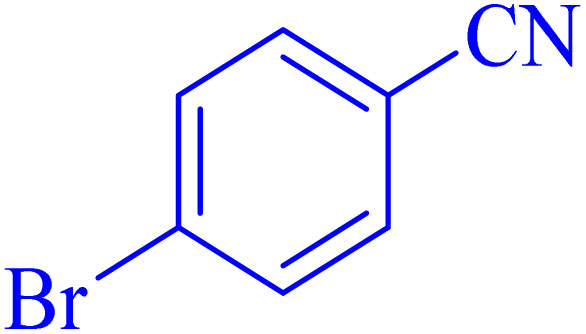	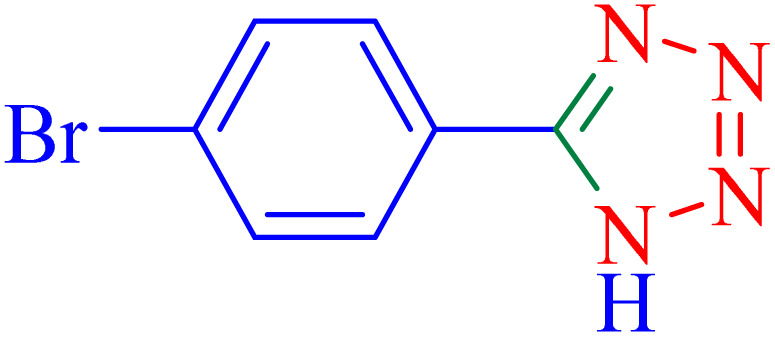	12	92	266–267	266–267 (ref. 7)
7	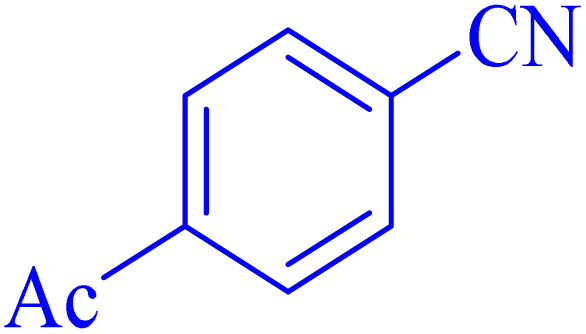	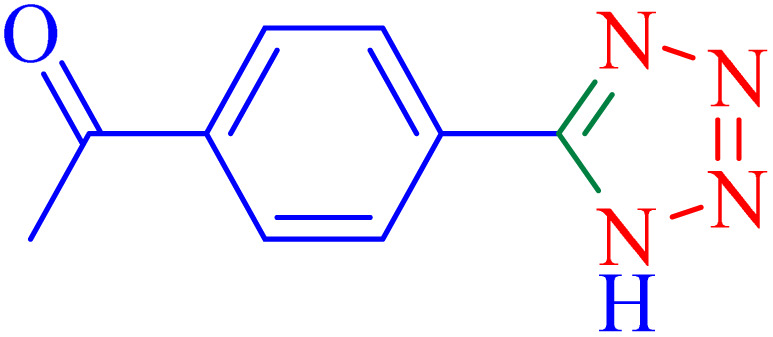	30	93	173–176	173–176 (ref. 7)
8	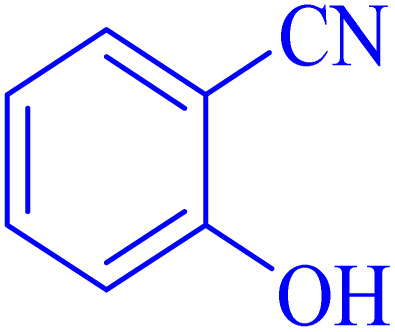	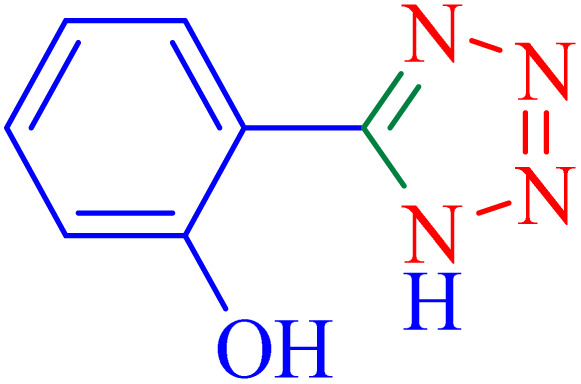	11	89	224–227	223–225 (ref. 49)
9	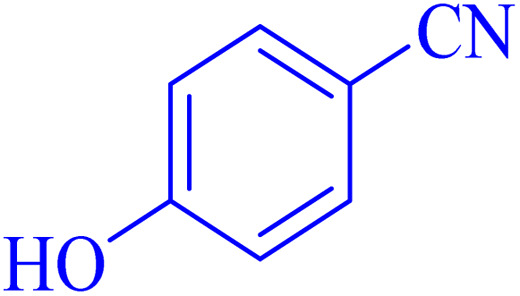	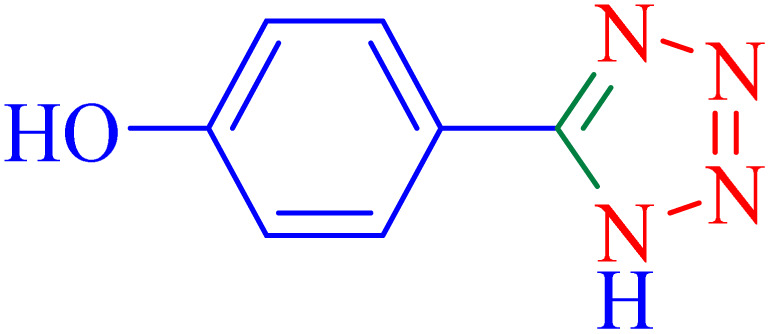	9	95	233–235	233–235 (ref. 7)
10	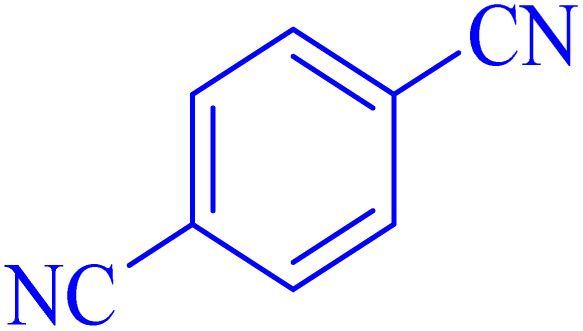	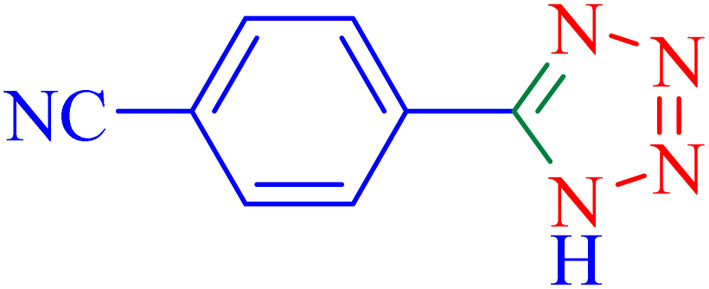	10.5	84	255–258	254–257 (ref. 7)

a Isolated yield.

The difference in reaction times between benzonitrile (8 hours) and 4-acetylbenzonitrile (30 hours) in tetrazole formation can be attributed to electronic effects exerted by the acetyl substituent. The acetyl group (–COCH_3_), being an electron-withdrawing group (EWG), increases the electrophilicity of the nitrile carbon through resonance and inductive effects, theoretically enhancing its reactivity toward nucleophilic attack by the azide ion. However, the longer reaction time observed for 4-acetylbenzonitrile suggests additional factors influencing the reaction kinetics. The acetyl group may stabilize the imidoyl azide intermediate formed during the reaction, slowing down its cyclization step. While EWGs facilitate nitrile activation, they might introduce steric or electronic hindrance during the subsequent steps. The acetyl group could promote side reactions under prolonged heating conditions, such as decomposition of the tetrazole product, which could reduce overall efficiency. In summary, while electron-withdrawing substituents generally enhance nitrile reactivity, the extended reaction time for 4-acetylbenzonitrile is likely due to a combination of intermediate stabilization and side reactions under the specific conditions used in this study. This highlights the nuanced interplay between electronic effects and reaction kinetics in tetrazole synthesis.

## 6. Proposed mechanism for the synthesis of tetrazoles

**Scheme 4:**
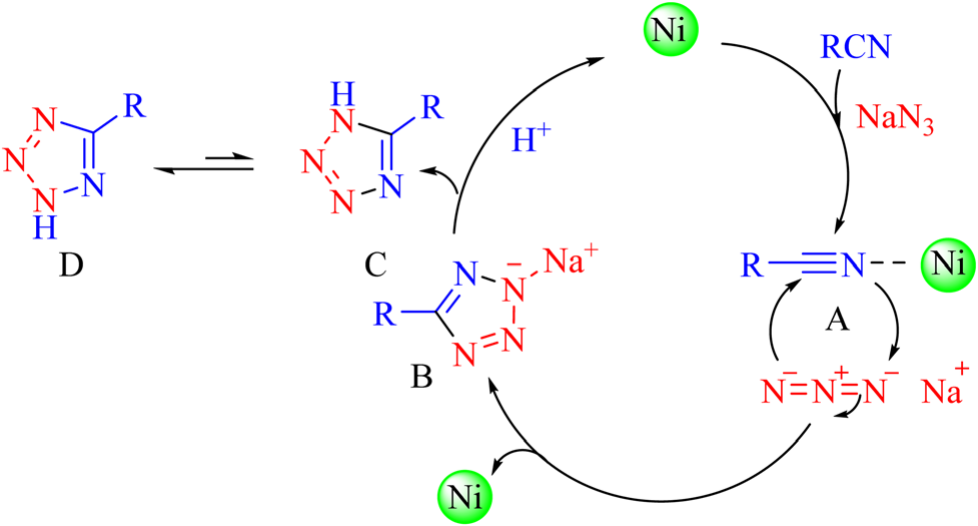
Proposed mechanism for the synthesis of tetrazoles catalyzed by Ni–ascorbic acid MOF.

A plausible mechanism is shown in Scheme 4.^50–54^ The nitrile compound is activated by the Ni–ascorbic acid MOF catalyst (A). This activation may involve coordination of the nitrile nitrogen to a Ni center, increasing the electrophilicity of the nitrile carbon. The [2 + 3] cycloaddition between the C≡N bond of the nitrile compound and the azide ion proceeds to form an intermediate B, which, upon acidic work-up, transforms into a mixture of tautomers (C, D), ultimately favoring the formation of the more stable 5-substituted 1*H*-tetrazole (D).

## 7. Recycling ability and hot filtration test of Ni–ascorbic acid MOF

**Fig. 8:**
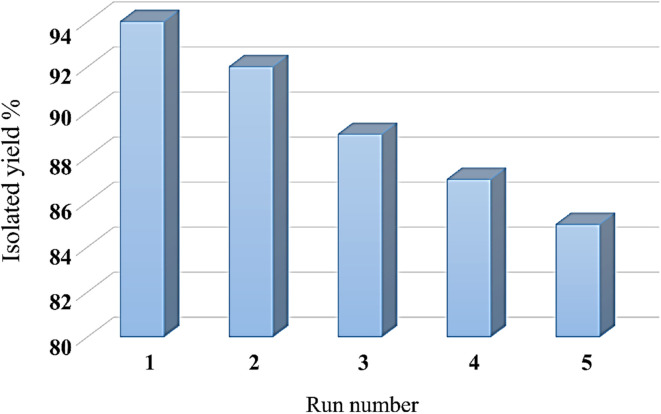
Recyclability of Ni–ascorbic acid MOF catalyst.

The recyclability of the Ni–ascorbic acid MOF was checked in the reaction of NaN_3_ and benzonitrile. Fig. 8 indicates that Ni–ascorbic acid MOF can be reused 5 times without significantly reducing its performance. Also, a hot filtration test was performed for Ni–ascorbic acid MOF, where Ni leaching into the solution or framework degradation was not observed. The proof of non-washing of the active species was confirmed by AAS (Atomic Absorption Spectroscopy). The AAS analysis of the Ni–ascorbic acid MOF recycled catalyst revealed a nickel concentration of 632.4 ppm, demonstrating effective retention of the active metal after recovery and reuse. This result confirms the structural integrity of the MOF framework in preserving nickel species during catalytic cycles, a critical factor for maintaining performance in successive applications.

**Fig. 9:**
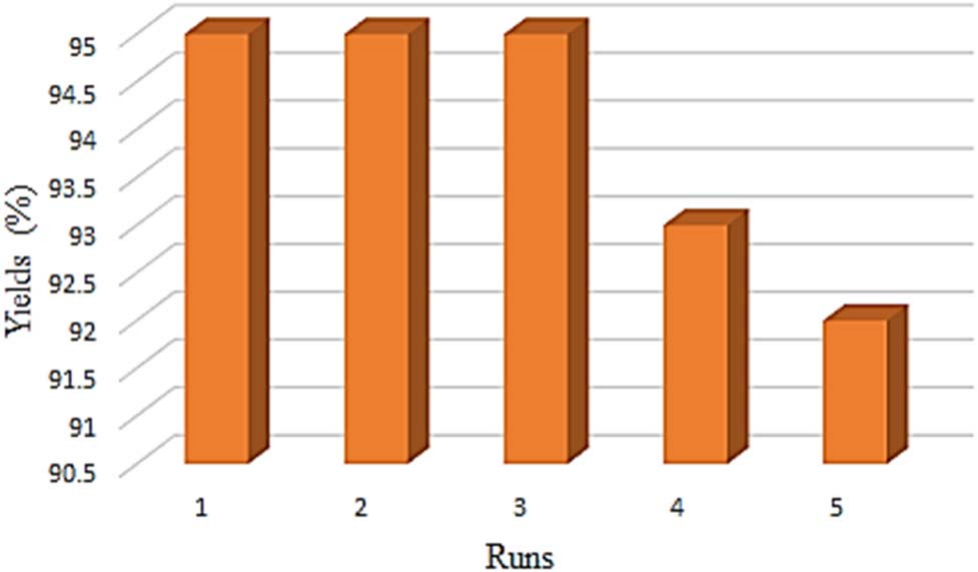
Recyclability of the BNPs@SiO_2_-TPPTSA in the synthesis of 5-substituted 1*H*-tetrazole.

**Fig. 10:**
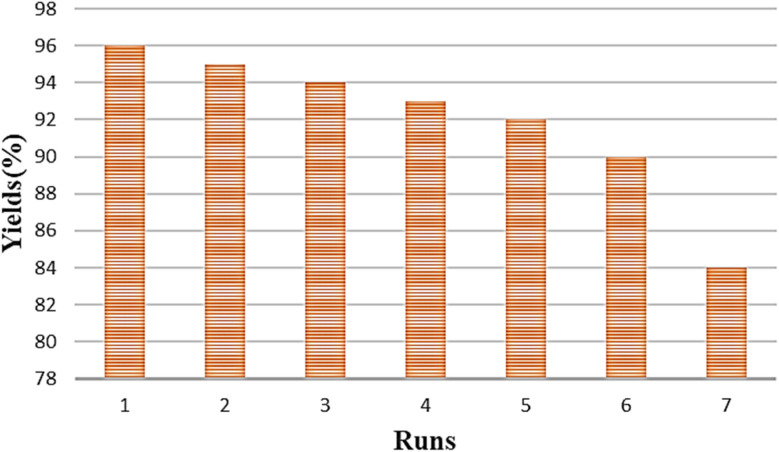
Recyclability of the Cu-Amd-RGO in the synthesis of 5-substituted 1*H*-tetrazole.

The comparison for the recovery of Ni–ascorbic acid MOF catalyst for the synthesis of 5-substituted 1*H*-tetrazole with the other catalysts, such as BNPs@SiO_2_-TPPTSA and Cu-Amd-RGO, is shown in Fig. 9 and 10.^55,56^

When considering Ni-MOF catalysts that interact with ascorbic acid, stability becomes a critical factor that determines their practical utility. This consideration is particularly relevant for Ni-MOF systems intended for ascorbic acid catalysis, as the reducing properties of ascorbic acid could potentially alter the metal centres or coordination environments within the MOF structure over time.

Despite these potential concerns, properly designed MOF-based composite catalysts can demonstrate remarkable stability. For instance, Deng *et al.* reported that their composite catalyst-maintained stability “after at least 4 recycles”.^57^ Similarly, Ni–ascorbic acid MOF recycling was possible up to 5 steps without a significant reduction in yield. This finding suggests that while stability is a legitimate concern for MOFs in the presence of reactive substrates like ascorbic acid, careful design of the catalyst architecture can help maintain structural integrity and catalytic performance over multiple cycles.

**Fig. 11:**
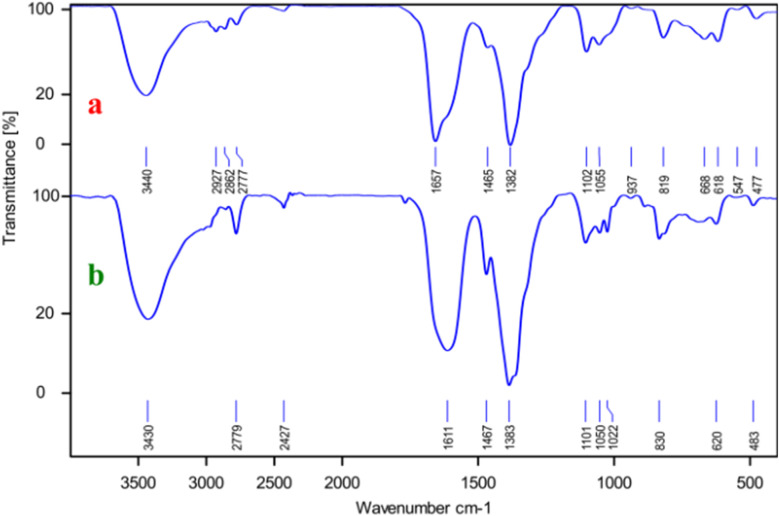
FT-IR spectra of Ni–ascorbic acid MOF before (a) and after (b) the reaction.

The stability analysis of Ni–ascorbic acid MOF was investigated using FT-IR spectroscopy. After repeated use (b) compared to the fresh catalyst (a). This finding indicates that the Ni–ascorbic acid MOF maintained its structural features throughout its operational cycles. The absence of significant shifts in the absorption bands suggests that both the chemical environment and the active sites of the catalyst were preserved, thereby ensuring consistent catalytic performance (Fig. 11).

**Fig. 12:**
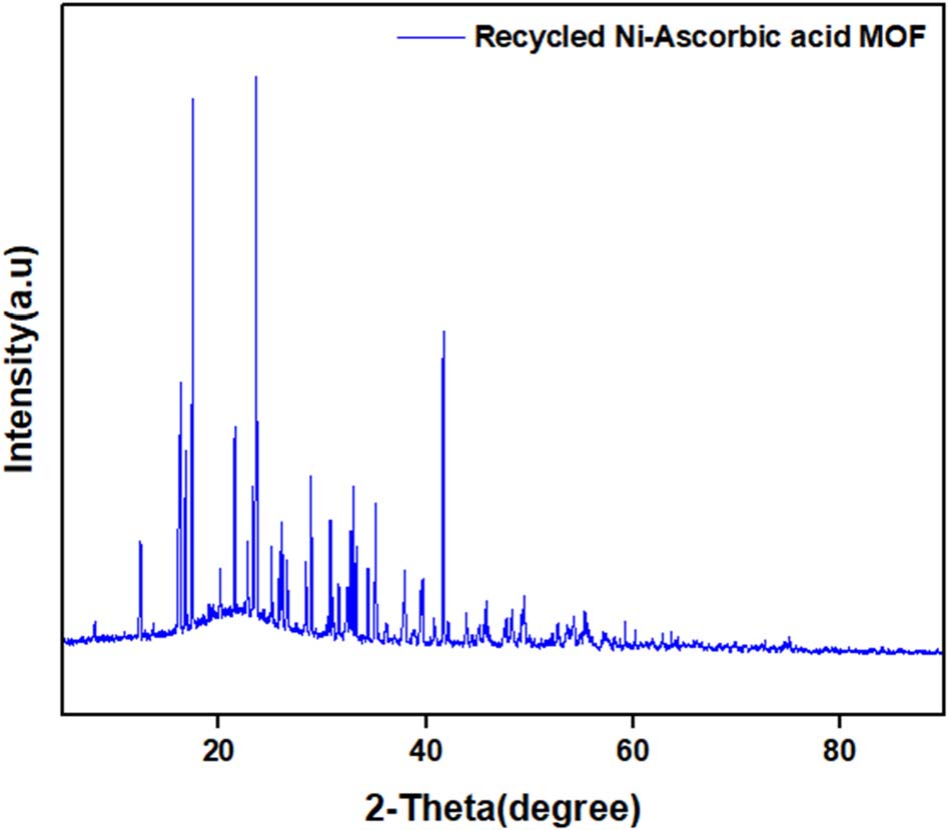
XRD spectra of Ni–ascorbic acid MOF after the reaction.

XRD analysis of the recycled Ni–ascorbic acid MOF shows that its structure remained largely unchanged compared to the unused catalyst, and the structural integrity was well maintained after multiple catalytic cycles (Fig. 12).

**Fig. 13:**
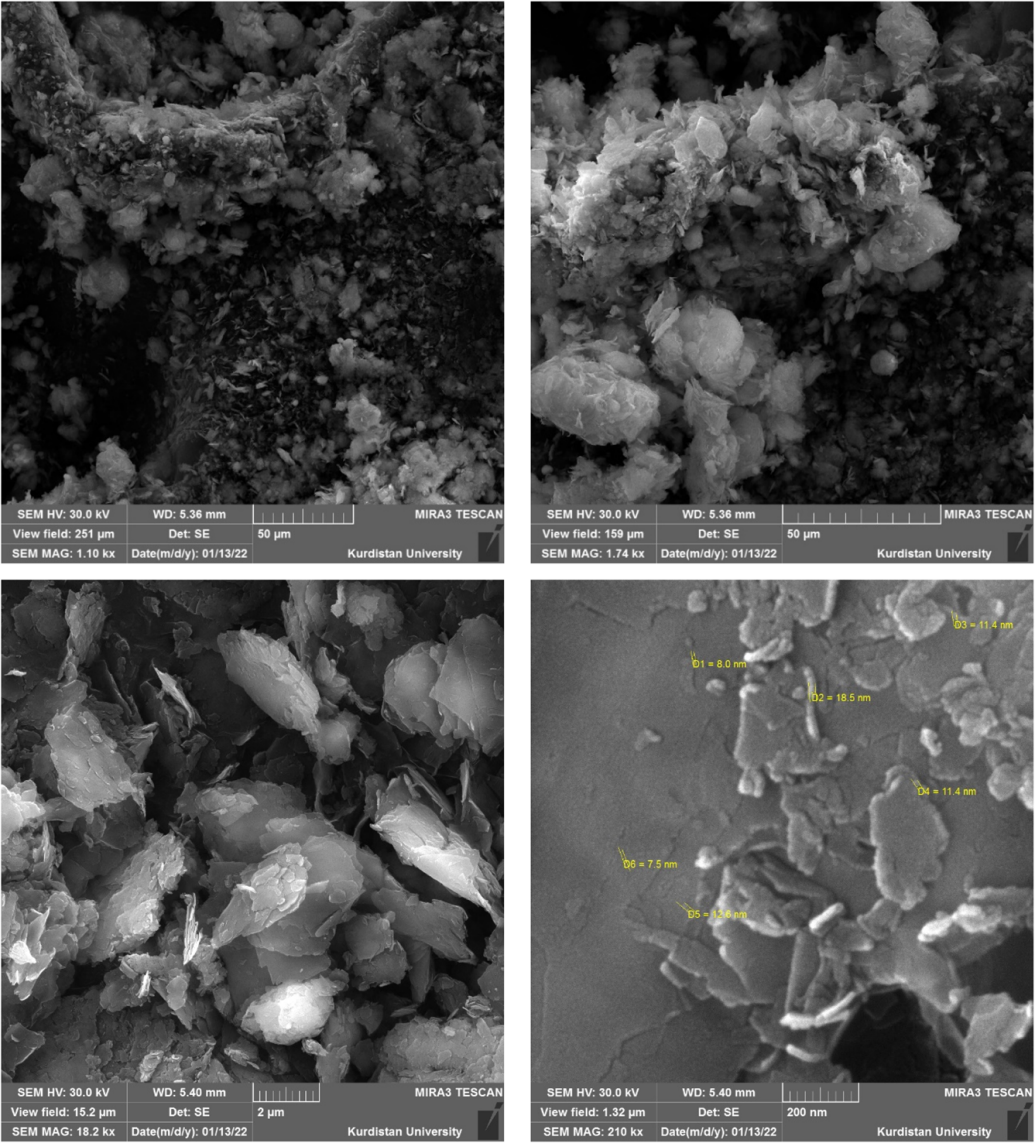
FE-SEM analysis of the recovered Ni-ascorbic acid MOF.

The Ni–ascorbic acid MOF morphology was analyzed using FE-SEM after repeated use, and the results showed that the morphology had not changed significantly (Fig. 13). This observation indicates that the catalyst exhibits high structural stability, even after numerous reaction cycles. The minimal changes in morphology suggest that the active sites and overall surface structure of the catalyst were preserved, which is crucial for maintaining its catalytic performance over time.

**Fig. 14:**
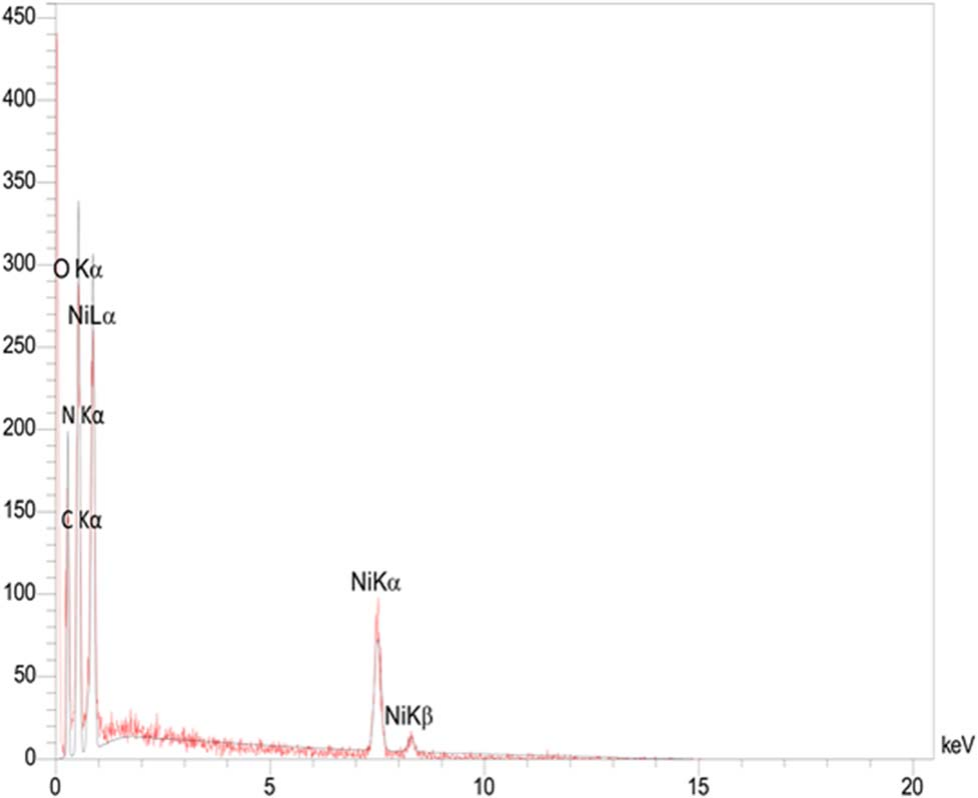
EDX analysis of the recovered Ni–ascorbic acid MOF.

The recovery of the Ni–ascorbic acid MOF catalyst is a crucial step to ensure its long-term application and sustainability. EDX spectra reveal the presence of nickel, oxygen, carbon, and nitrogen (Fig. 14), indicating that this catalyst is an excellent candidate for successful recovery and reuse. The structural integrity and compositional consistency of the catalyst, even after multiple cycles, indicate effective recovery. This capability not only increases its economic viability but also reduces waste and is in line with environmentally friendly approaches in catalytic applications.

**Fig. 15:**
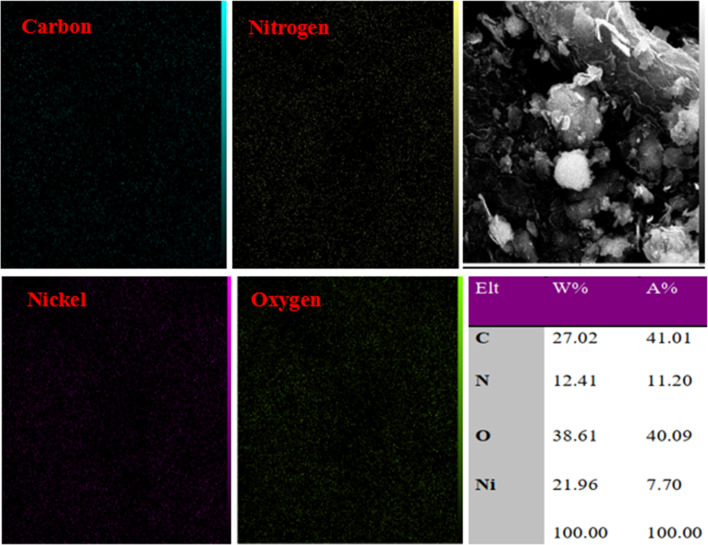
Elemental mapping analysis of the recovered Ni–ascorbic acid MOF.

The elemental mapping analysis of the recycled Ni–ascorbic acid MOF catalyst demonstrated a preserved uniformity in the spatial distribution of carbon, oxygen, nitrogen, and nickel elements, closely corresponding to the distribution patterns observed in the fresh catalyst (Fig. 15). This observation suggests the maintenance of the structural integrity of the ascorbic acid-derived organic framework over successive catalytic cycles.

## 8. Comparison

Finally, the current protocol was compared with previously reported catalysts (Table 6). The results indicated superior performance for the formation of all products when using the Ni–ascorbic acid MOF. This enhanced performance is attributed to the synergistic effect between nickel and the coordinated sites within the Ni–ascorbic acid MOF catalytic system.

**Table 6:** Comparison of the synthesis of 5-substituted 1*H*-tetrazoles and oxidation of sulfides

Entry	Synthesis	Catalyst	Time (h)	Yield^a^ (%)	Ref.
1	5-Substituted 1*H*-tetrazole	Amberlyst-15	12	91	58
2	5-Substituted 1*H*-tetrazole	Ln(OTf)_3_-SiO_2_	12	88	59
3	5-Substituted 1*H*-tetrazole	Fe_3_O_4_@AMPD@Ni	3	93	60
4	5-Substituted 1*H*-tetrazole	Cu (OAc)_2_	12	90	61
5	5-Substituted 1*H*-tetrazole	Ni–ascorbic acid MOF	8	97	This work
6	Sulfoxidation	ZrO-SB-APT@MCM-41	0.75	91	46
7	Sulfoxidation	DSA@MNPs	6	98	62
8	Sulfoxidation	Ni-dithizone@boehmite	1.33	96	47
9	Sulfoxidation	Copper–Schiff base complex	9	82	38
10	Sulfoxidation	Ni–ascorbic acid MOF	0.58	95	This work

a Isolated yield.

## 9. Conclusion

In summary, a novel and heterogeneous Ni–ascorbic acid MOF catalyst was successfully synthesized using commercially available ascorbic acid and nickel nitrate solutions as starting materials. This catalyst demonstrated high activity for the oxidation of sulfides to sulfoxides and the [2 + 3] cycloaddition synthesis of 5-substituted 1*H*-tetrazoles. Key advantages of the Ni–ascorbic acid MOF include high yields, short reaction times, and environmentally friendly conditions. Additionally, the catalyst was easily separated from the reaction mixture and reused up to five times, showcasing its remarkable stability and reusability.

## Ethical statement

This article does not include experiments involving human tissue.

## Data availability

All data are available in the article and the ESI.†

## Author contributions

Mostafa Koolivand. Did the experimental works, methodology, software, writing, review & and editing. Zahra Moradi. Did the investigation and data curation. Mohsen Nikoorazm. Supervised the research project, writing review & and editing, and is the primary corresponding author of the manuscript. Arash Ghorbani-Choghamarani. Supervised the research project.

## Conflicts of interest

All co-authors have seen and agreed with the manuscript's contents, and there are no conflicts of interest or competing interests.

## Supplementary Material

RA-015-D5RA00355E-s001
