# Supplementary material for: Preparation of a Ni–ascorbic acid MOF as a recyclable catalyst for the synthesis of sulfoxides and tetrazoles
Source: RSC Adv. 2025 May 12;15(20):15748–62. doi: 10.1039/d5ra00355e (PMC12068477; doi:10.1039/d5ra00355e)
Supplement: RA-015-D5RA00355E-s001 [file RA-015-D5RA00355E-s001.pdf]

Supplementary Information (SI) for RSC Advances.
This journal is © The Royal Society of Chemistry 2025

Preparation of Ni-Ascorbic acid MOF as a recyclable catalyst for the synthesis of sulfoxides and tetrazoles

Mostafa Koolivand [a], Zahra Moradi [a], Mohsen Nikoorazm [a,*], Arash Gtahorbani-Choghamarani [b]

[a] Department of Chemistry, Faculty of Science, Ilam University, Ilam, Iran.

[b] Department of Organic Chemistry, Bu-Ali Sina University, 6517838683, Hamedan, Iran.

Abstract

This study introduces a novel, cost-effective, and environmentally friendly approach for synthesizing a heterogeneous Ni-Ascorbic acid metal-organic framework (Ni-Ascorbic acid MOF) catalyst via a hydrothermal method. The catalyst was prepared by combining nickel nitrate ($Ni(NO_3)_2 \cdot 6H_2O$) and ascorbic acid ($C_6H_8O_6$) in DMF. Comprehensive characterization of the synthesized Ni-Ascorbic acid MOF was performed using FT-IR, XRD, EDX, BET, SEM, TGA/DSC, and AAS techniques. These analyses revealed that the catalyst exhibits a spherical microsphere morphology with high crystallinity, a specific surface area of 16.62 m^2/g, a pore diameter of 19.52 nm, and excellent thermal stability. The catalytic performance of Ni-Ascorbic acid MOF was investigated in two distinct reactions including the selective oxidation of sulfides to sulfoxides and the synthesis of 5-substituted 1*H*-tetrazoles. Under optimized reaction conditions, the catalyst demonstrated high efficiency with product yields ranging from moderate to excellent across various substrates. Furthermore, the catalyst exhibited remarkable recyclability, maintaining its activity over five consecutive cycles without significant leaching of nickel species, as confirmed by hot filtration tests. These findings underscore the potential of Ni-Ascorbic acid MOF as a sustainable and robust catalyst for diverse organic transformations.

* Address correspondence to M. Nikoorazm, E-mail address: m.nikorazm@ilam.ac.ir & e_nikoorazm@yahoo.com.

^{1}H NMR spectral data

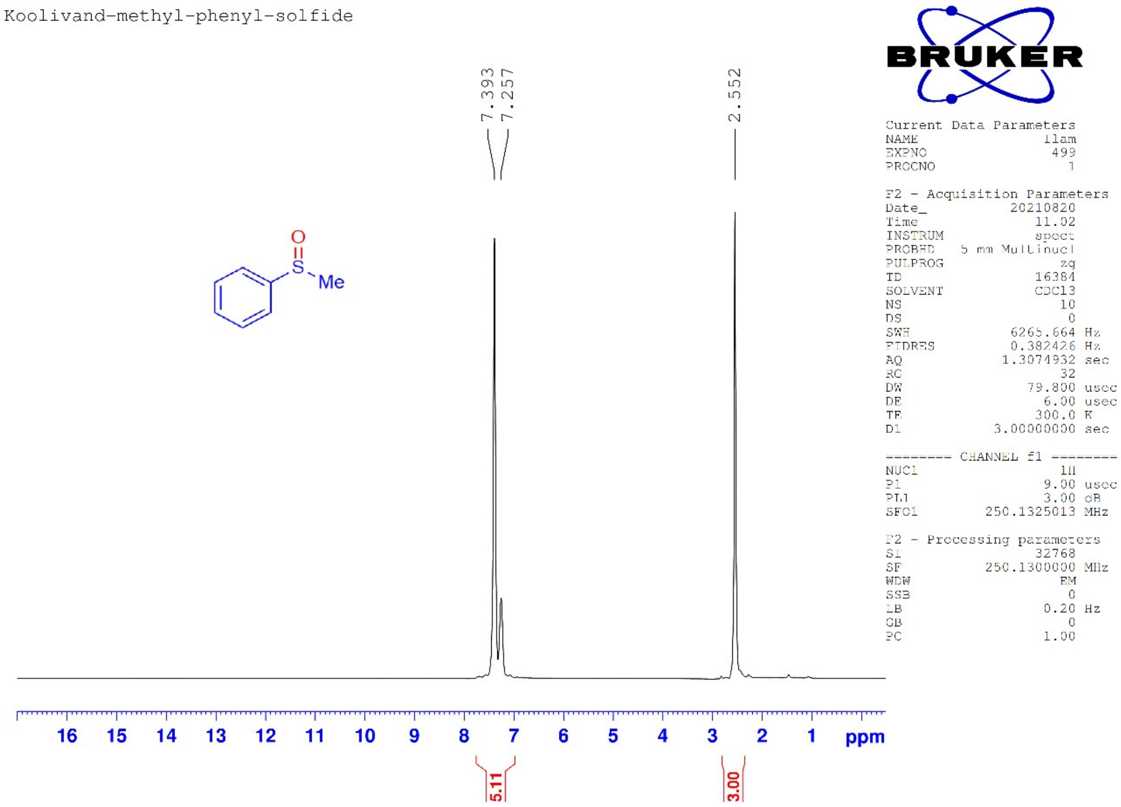

Figure S1.

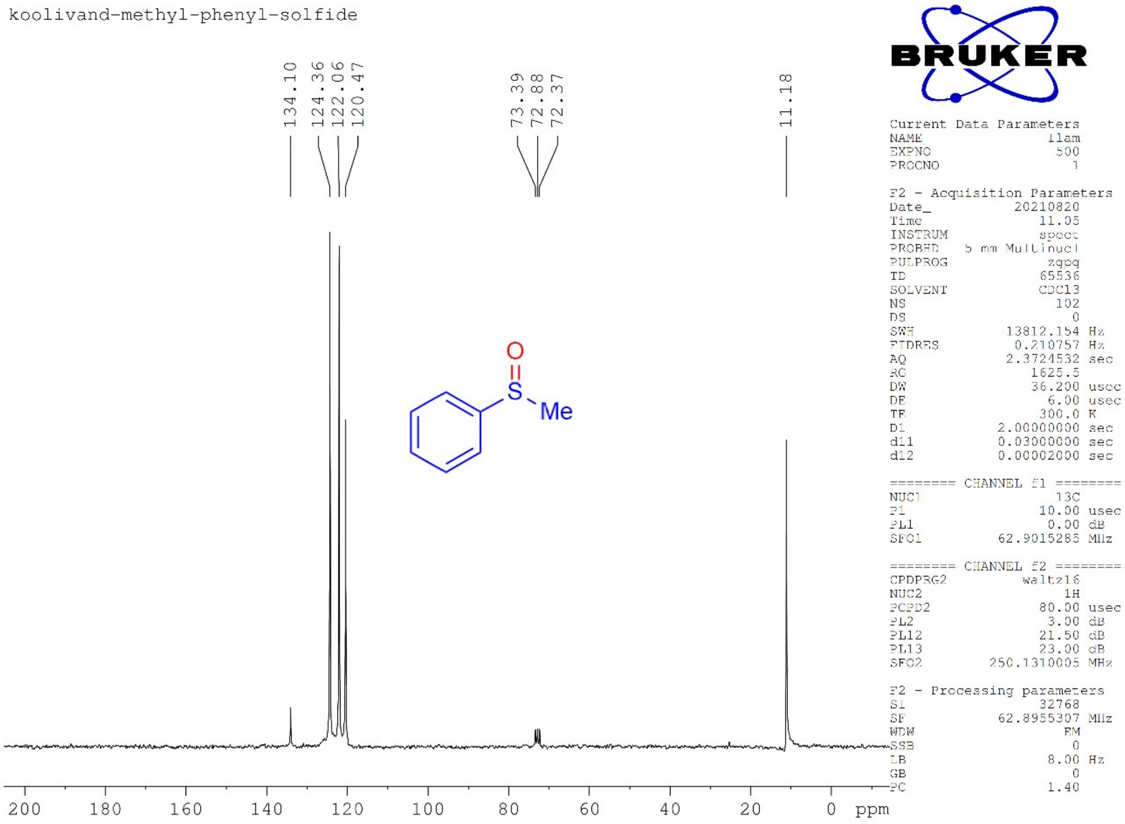
koolivand-methyl-phenyl-solfide
134.10
124.36
122.06
120.47
73.39
72.88
72.37
11.18
S
O
Me
BRUKER
Current Data Parameters
NAME 11am
EXPNO 500
PROCNO 1
F2 - Acquisition Parameters
Date_ 20210820
Time 11.05
INSTRUM spect
PROBHD 5 mm Multinucl
PULPROG zgpg
TD 65536
SOLVENT CDCl3
NS 102
DS 0
SWH 13812.154 Hz
FIDRES 0.210757 Hz
AQ 2.3724532 sec
RG 1625.5
DW 36.200 usec
DE 6.00 usec
TE 300.0 K
D1 2.00000000 sec
d11 0.03000000 sec
d12 0.00002000 sec
======== CHANNEL f1 ========
NUC1 13C
P1 10.00 usec
PL1 0.00 dB
SFO1 62.9015285 MHz
======== CHANNEL f2 ========
CPDPRG2 waltz16
NUC2 1H
PCPD2 80.00 usec
PL2 3.00 dB
PL12 21.50 dB
PL13 23.00 dB
SFO2 250.1310005 MHz
F2 - Processing parameters
SI 32768
SF 62.8955307 MHz
WDW EM
SSB 0
LB 8.00 Hz
GB 0
PC 1.40
200 180 160 140 120 100 80 60 40 20 0 ppm

Figure S2.

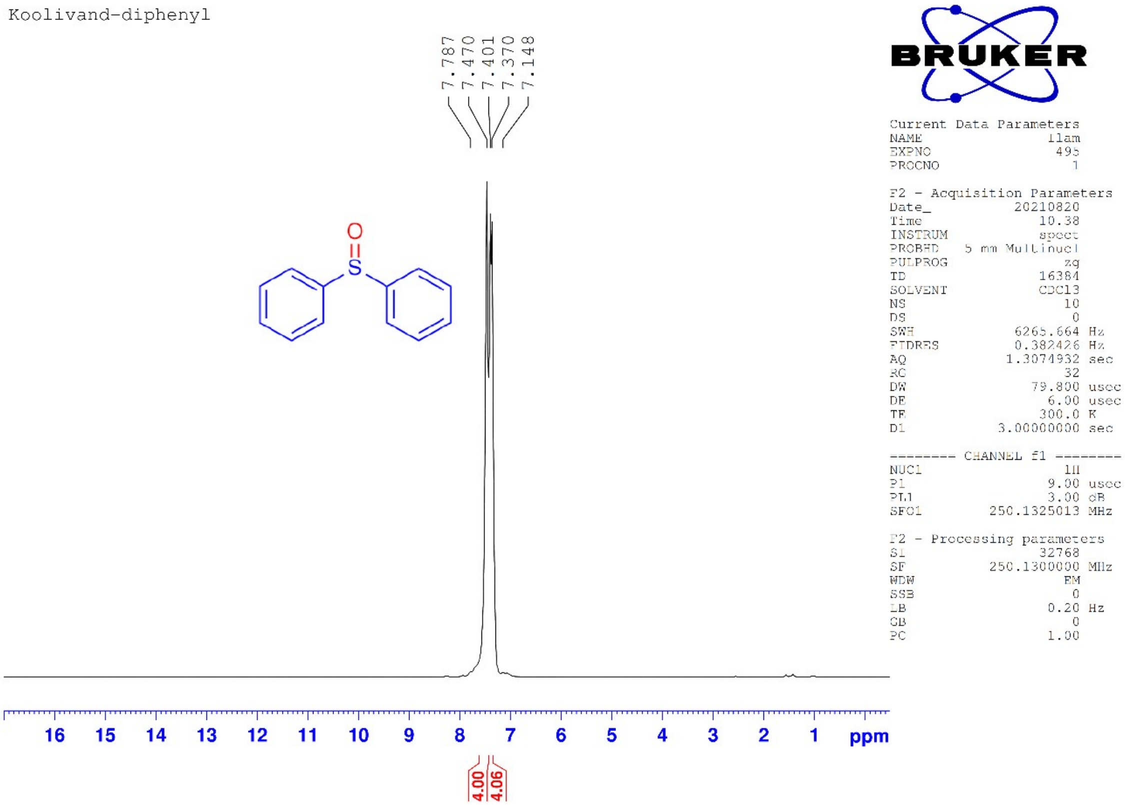
Koolivand-diphenyl
7.787
7.470
7.401
7.370
7.148
O
S
BRUKER
Current Data Parameters
NAME 11am
EXPNO 495
PROCNO 1
F2 - Acquisition Parameters
Date_ 20210820
Time 10.38
INSTRUM spect
PROBHD 5 mm Multinucl
PULPROG zg
TD 16384
SOLVENT CDCl3
NS 10
DS 0
SWH 6265.664 Hz
FIDRES 0.382426 Hz
AQ 1.3074932 sec
RG 32
DW 79.800 usec
DE 6.00 usec
TE 300.0 K
D1 3.00000000 sec
-------- CHANNEL f1 --------
NUC1 1H
P1 9.00 usec
PL1 3.00 dB
SFO1 250.1325013 MHz
F2 - Processing parameters
SI 32768
SF 250.1300000 MHz
WDW EM
SSB 0
LB 0.20 Hz
GB 0
PC 1.00
16 15 14 13 12 11 10 9 8 7 6 5 4 3 2 1 ppm
4.00
4.06

Figure S3.

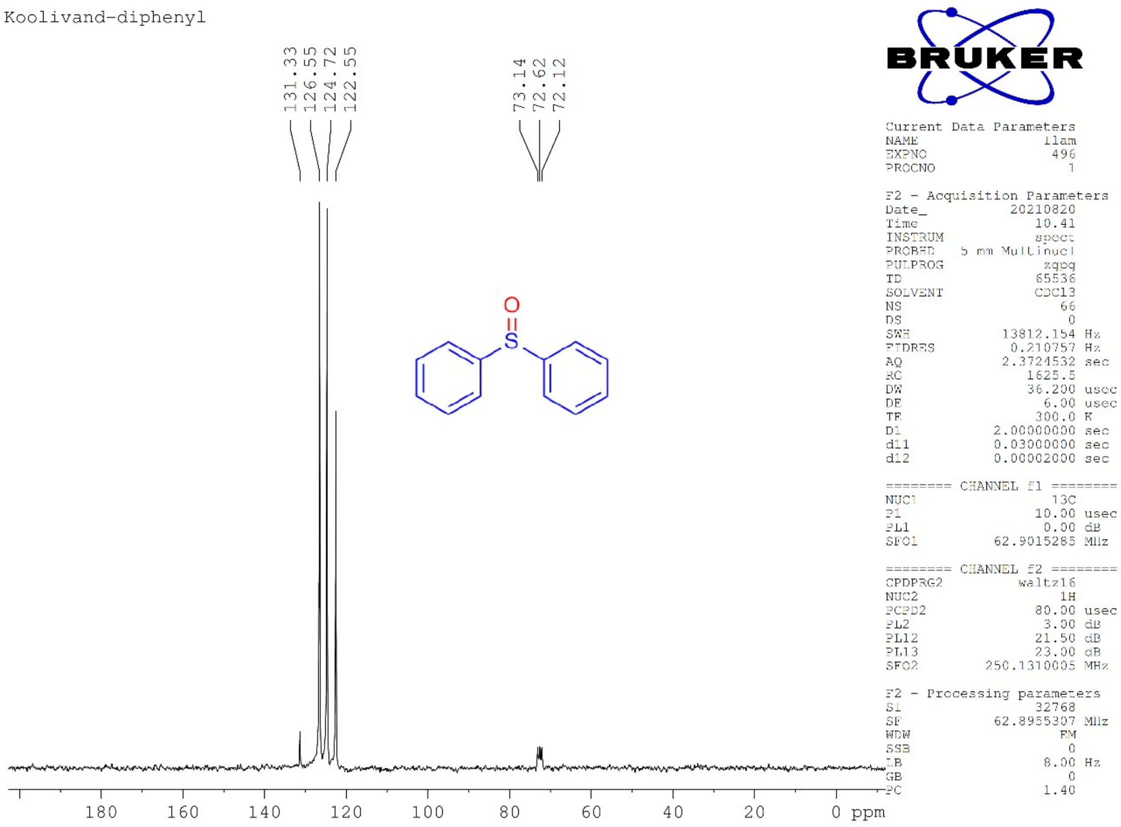
Koolivand-diphenyl
131.33
126.55
124.72
122.55
73.14
72.62
72.12
O
S
BRUKER
Current Data Parameters
NAME llam
EXPNO 496
PROCNO 1
F2 - Acquisition Parameters
Date_ 20210820
Time 10.41
INSTRUM spect
PROBHD 5 mm Multinucl
PULPROG zgpg
TD 65536
SOLVENT CDCl3
NS 66
DS 0
SWH 13812.154 Hz
FIDRES 0.210757 Hz
AQ 2.3724532 sec
RG 1625.5
DW 36.200 usec
DE 6.00 usec
TE 300.0 K
D1 2.00000000 sec
d11 0.03000000 sec
d12 0.00002000 sec
======== CHANNEL f1 ========
NUC1 13C
P1 10.00 usec
PL1 0.00 dB
SFO1 62.9015285 MHz
======== CHANNEL f2 ========
CPDPRG2 waltz16
NUC2 1H
PCPD2 80.00 usec
PL2 3.00 dB
PL12 21.50 dB
PL13 23.00 dB
SFO2 250.1310005 MHz
F2 - Processing parameters
SI 32768
SF 62.8955307 MHz
WDW EM
SSB 0
LB 8.00 Hz
GB 0
PC 1.40
180
160
140
120
100
80
60
40
20
0 ppm

Figure S4.

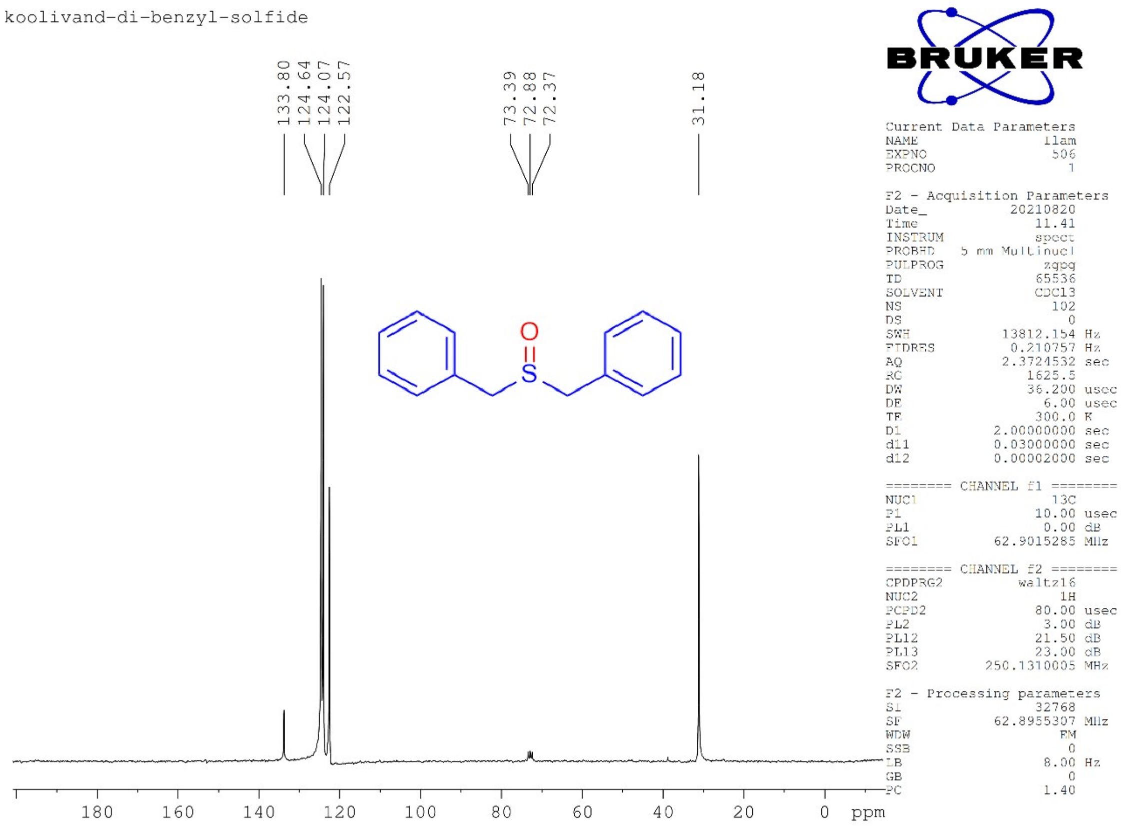

koolivand-di-benzyl-solfide
133.80
124.64
124.07
122.57
73.39
72.88
72.37
31.18
O
S
BRUKER
Current Data Parameters
NAME 11am
EXPNO 506
PROCNO 1
F2 - Acquisition Parameters
Date_ 20210820
Time 11.41
INSTRUM spect
PROBHD 5 mm Multinucl
PULPROG zgpg
TD 65536
SOLVENT CDCl3
NS 102
DS 0
SWH 13812.154 Hz
FIDRES 0.210757 Hz
AQ 2.3724532 sec
RG 1625.5
DW 36.200 usec
DE 6.00 usec
TE 300.0 K
D1 2.00000000 sec
d11 0.03000000 sec
d12 0.00002000 sec
======== CHANNEL f1 ========
NUC1 13C
P1 10.00 usec
PL1 0.00 dB
SFO1 62.9015285 MHz
======== CHANNEL f2 ========
CPDPRG2 waltz16
NUC2 1H
PCPD2 80.00 usec
PL2 3.00 dB
PL12 21.50 dB
PL13 23.00 dB
SFO2 250.1310005 MHz
F2 - Processing parameters
SI 32768
SF 62.8955307 MHz
WDW EM
SSB 0
LB 8.00 Hz
GB 0
PC 1.40
180
160
140
120
100
80
60
40
20
0
ppm

Figure S5.

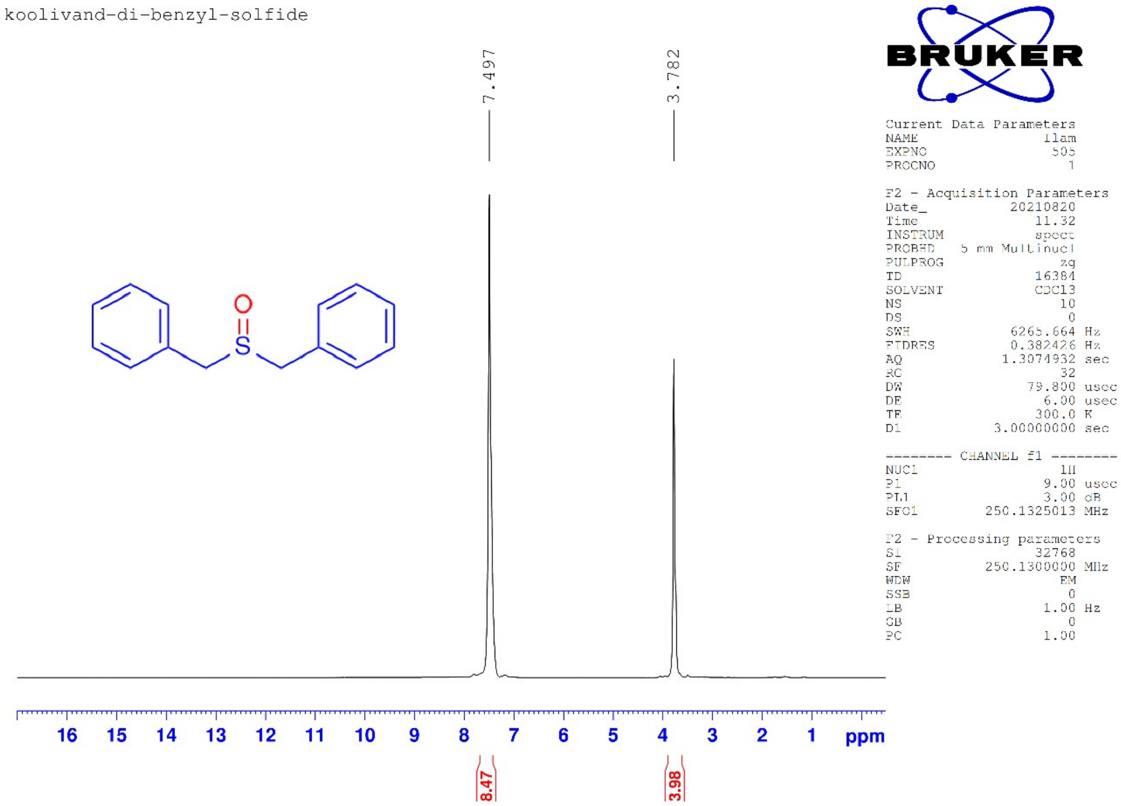
koolivand-di-benzyl-solfide
7.497
3.782
O
S
BRUKER
Current Data Parameters
NAME 11am
EXPNO 505
PROCNO 1
F2 - Acquisition Parameters
Date_ 20210820
Time 11.32
INSTRUM spect
PROBHD 5 mm Multinucl
PULPROG zg
TD 16384
SOLVENT CDCl3
NS 10
DS 0
SWH 6265.664 Hz
FIDRES 0.382426 Hz
AQ 1.3074932 sec
RG 32
DW 79.800 usec
DE 6.00 usec
TE 300.0 K
D1 3.00000000 sec
-------- CHANNEL f1 --------
NUC1 1H
P1 9.00 usec
PL1 3.00 dB
SFO1 250.1325013 MHz
F2 - Processing parameters
SI 32768
SF 250.1300000 MHz
WDW EM
SSB 0
LB 1.00 Hz
GB 0
PC 1.00
16 15 14 13 12 11 10 9 8 7 6 5 4 3 2 1 ppm
8.47
3.98

Figure S6.

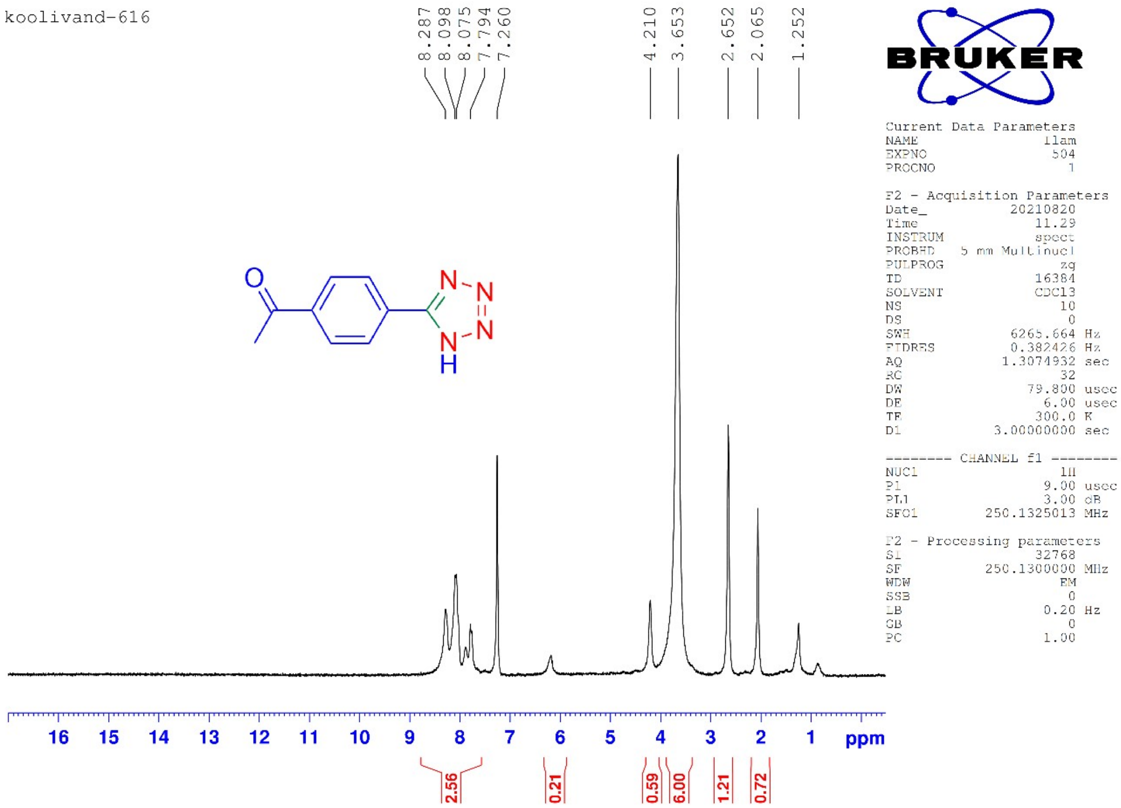
koolivand-616
8.287
8.098
8.075
7.794
7.260
4.210
3.653
2.652
2.065
1.252
Current Data Parameters
NAME Ilam
EXPNO 504
PROCNO 1
F2 - Acquisition Parameters
Date_ 20210820
Time 11.29
INSTRUM spect
PROBHD 5 mm Multinucl
PULPROG zg
TD 16384
SOLVENT CDCl3
NS 10
DS 0
SWH 6265.664 Hz
FIDRES 0.382426 Hz
AQ 1.3074932 sec
RG 32
DW 79.800 usec
DE 6.00 usec
TE 300.0 K
D1 3.00000000 sec
======== CHANNEL f1 ========
NUC1 1H
P1 9.00 usec
PL1 3.00 dB
SFO1 250.1325013 MHz
F2 - Processing parameters
SI 32768
SF 250.1300000 MHz
WDW EM
SSB 0
LB 0.20 Hz
GB 0
PC 1.00
16 15 14 13 12 11 10 9 8 7 6 5 4 3 2 1 ppm
2.56
0.21
0.59
6.00
1.21
0.72

Figure S7.

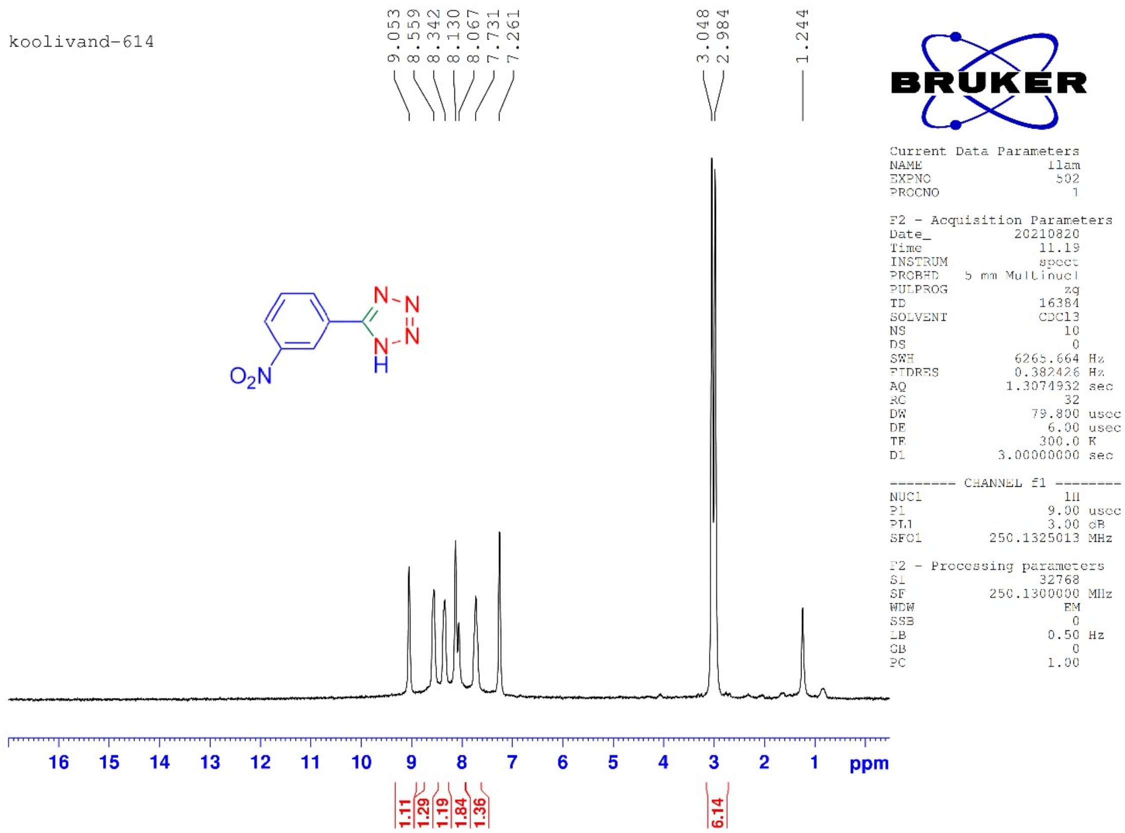

koolivand-614
9.053
8.559
8.342
8.130
8.067
7.731
7.261
3.048
2.984
1.244
BRUKER
Current Data Parameters
NAME Ilam
EXPNO 502
PROCNO 1
F2 - Acquisition Parameters
Date_ 20210820
Time 11.19
INSTRUM spect
PROBHD 5 mm Multinucl
PULPROG zg
TD 16384
SOLVENT CDCl3
NS 10
DS 0
SWH 6265.664 Hz
FIDRES 0.382426 Hz
AQ 1.3074932 sec
RG 32
DW 79.800 usec
DE 6.00 usec
TE 300.0 K
D1 3.00000000 sec
-------- CHANNEL f1 --------
NUC1 1H
P1 9.00 usec
PL1 3.00 dB
SFO1 250.1325013 MHz
F2 - Processing parameters
SI 32768
SF 250.1300000 MHz
WDW EM
SSB 0
LB 0.50 Hz
GB 0
PC 1.00
16 15 14 13 12 11 10 9 8 7 6 5 4 3 2 1 ppm
1.11
1.29
1.19
1.84
1.36
6.14

Figure S8.

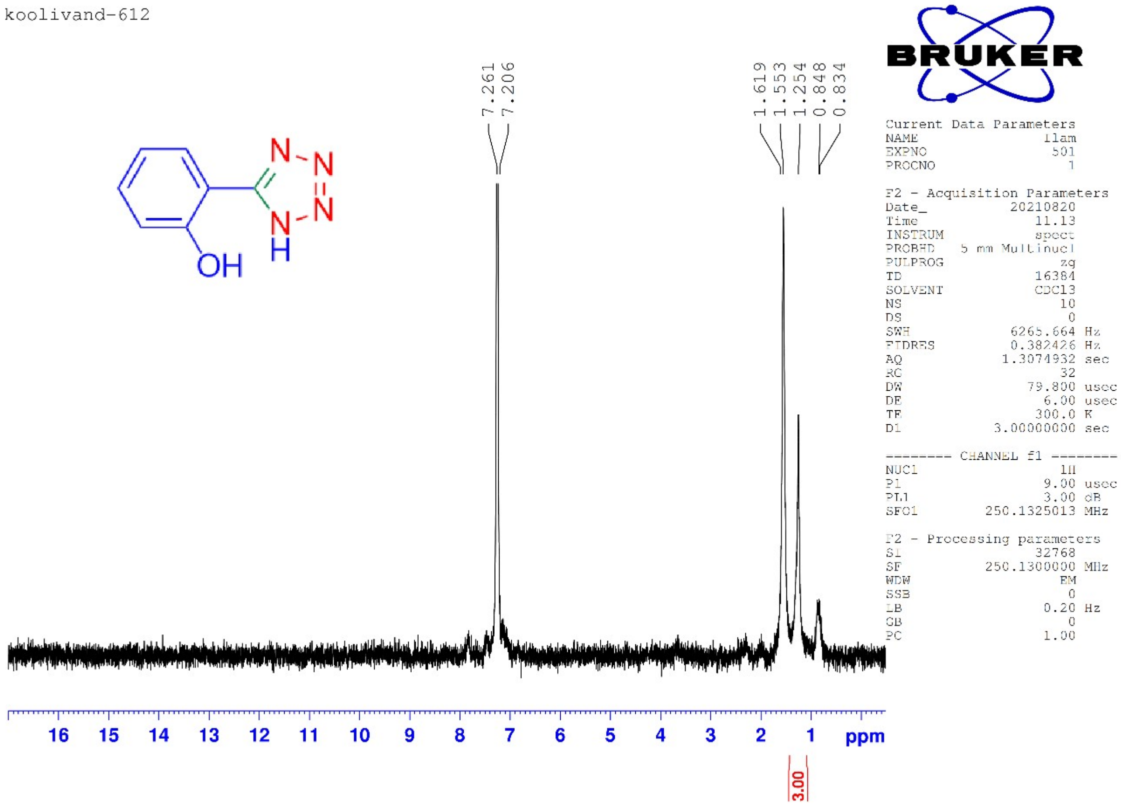
koolivand-612
7.261
7.206
1.619
1.553
1.254
0.848
0.834
OH
N
H
Current Data Parameters
NAME 11am
EXPNO 501
PROCNO 1
F2 - Acquisition Parameters
Date_ 20210820
Time 11.13
INSTRUM spect
PROBHD 5 mm Multinucl
PULPROG zg
TD 16384
SOLVENT CDCl3
NS 10
DS 0
SWH 6265.664 Hz
FIDRES 0.382426 Hz
AQ 1.3074932 sec
RG 32
DW 79.800 usec
DE 6.00 usec
TE 300.0 K
D1 3.00000000 sec
-------- CHANNEL f1 --------
NUC1 1H
P1 9.00 usec
PL1 3.00 dB
SFO1 250.1325013 MHz
F2 - Processing parameters
SI 32768
SF 250.1300000 MHz
WDW EM
SSB 0
LB 0.20 Hz
GB 0
PC 1.00
16 15 14 13 12 11 10 9 8 7 6 5 4 3 2 1 ppm
3.00

Figure S9.

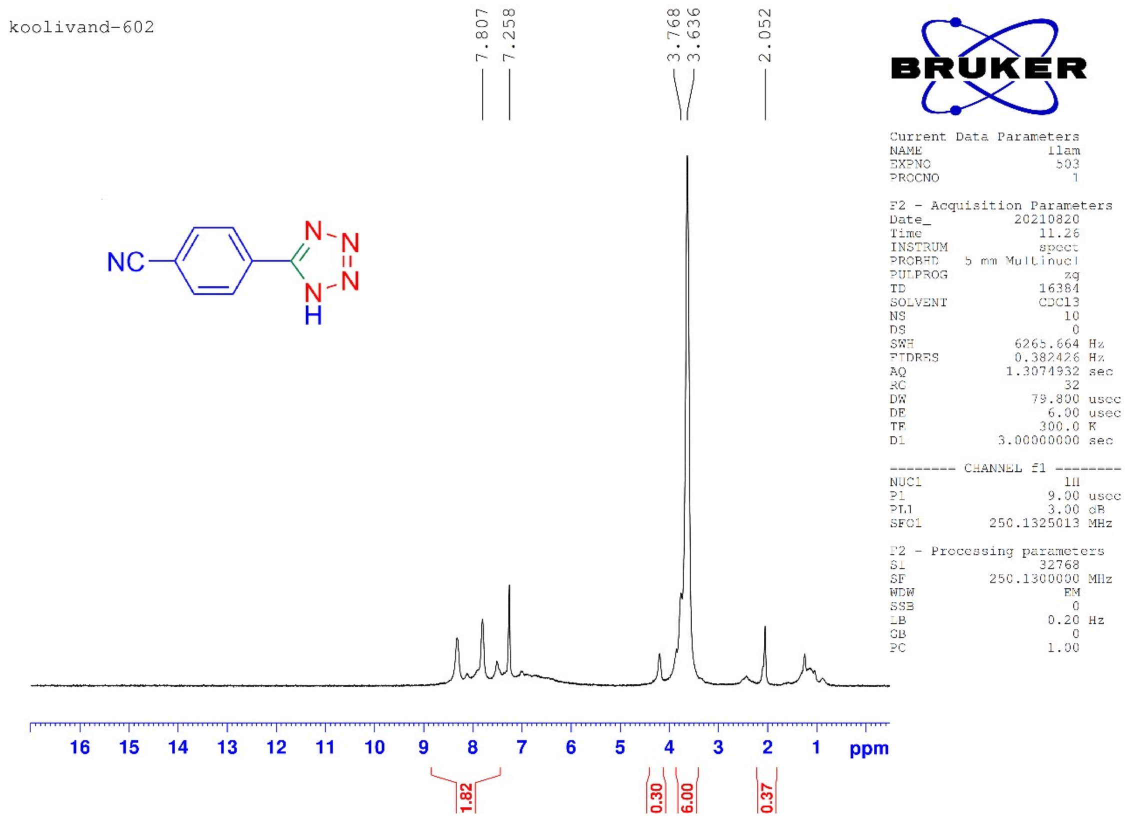
koolivand-602
7.807
7.258
3.768
3.636
2.052
NC
N
N
N
N
H
BRUKER
Current Data Parameters
NAME 11am
EXPNO 503
PROCNO 1
F2 - Acquisition Parameters
Date_ 20210820
Time 11.26
INSTRUM spect
PROBHD 5 mm Multinucl
PULPROG zg
TD 16384
SOLVENT CDCl3
NS 10
DS 0
SWH 6265.664 Hz
FIDRES 0.382426 Hz
AQ 1.3074932 sec
RG 32
DW 79.800 usec
DE 6.00 usec
TE 300.0 K
D1 3.00000000 sec
-------- CHANNEL f1 --------
NUC1 1H
P1 9.00 usec
PL1 3.00 dB
SFO1 250.1325013 MHz
F2 - Processing parameters
SI 32768
SF 250.1300000 MHz
WDW EM
SSB 0
LB 0.20 Hz
GB 0
PC 1.00
16 15 14 13 12 11 10 9 8 7 6 5 4 3 2 1 ppm
1.82
0.30
6.00
0.37

Figure S10.
